# Improving the Efficiency of Variationally Enhanced Sampling with Wavelet-Based Bias Potentials

**DOI:** 10.1021/acs.jctc.2c00197

**Published:** 2022-06-28

**Authors:** Benjamin Pampel, Omar Valsson

**Affiliations:** Max Planck Institute for Polymer Research, Ackermannweg 10, D-55128 Mainz, Germany

## Abstract

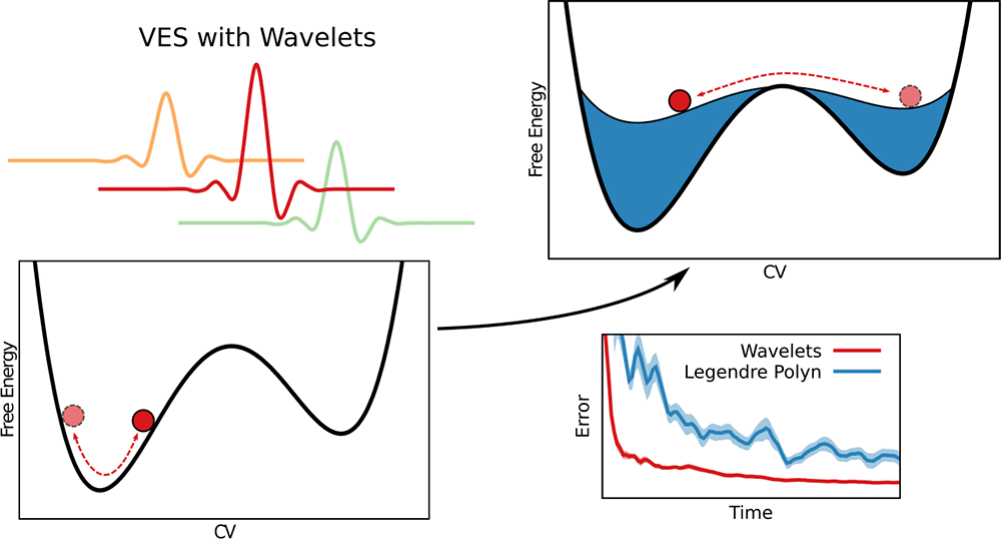

Collective variable-based enhanced sampling methods are routinely used on systems with metastable states, where high free energy barriers impede the proper sampling of the free energy landscapes when using conventional molecular dynamics simulations. One such method is variationally enhanced sampling (VES), which is based on a variational principle where a bias potential in the space of some chosen slow degrees of freedom, or collective variables, is constructed by minimizing a convex functional. In practice, the bias potential is taken as a linear expansion in some basis function set. So far, primarily basis functions delocalized in the collective variable space, like plane waves, Chebyshev, or Legendre polynomials, have been used. However, there has not been an extensive study of how the convergence behavior is affected by the choice of the basis functions. In particular, it remains an open question if localized basis functions might perform better. In this work, we implement, tune, and validate Daubechies wavelets as basis functions for VES. The wavelets construct orthogonal and localized bases that exhibit an attractive multiresolution property. We evaluate the performance of wavelet and other basis functions on various systems, going from model potentials to the calcium carbonate association process in water. We observe that wavelets exhibit excellent performance and much more robust convergence behavior than all other basis functions, as well as better performance than metadynamics. In particular, using wavelet bases yields far smaller fluctuations of the bias potential within individual runs and smaller differences between independent runs. Based on our overall results, we can recommend wavelets as basis functions for VES.

## 1 Introduction

A major problem impeding conventional molecular dynamics (MD) simulations is the so-called time scale or rare event problem. Often, the molecular process of interest occurs on a much longer time scale than one can simulate in practice; in other words, it is a rare event. Thus, the system stays in a metastable state during the simulation, and one does not observe transitions to other metastable states. Despite impressive developments in specialized hardware^1,2^ and MD codes^3,4^ that make very efficient usage of modern graphics processing units, it is unlikely that accessible time scales will increase significantly in the near future. The speedup of individual processing units has come to an end and high-performance computing relies on the usage of massive parallelization,^5^ and time is not easily parallelizable. Thus, there has been considerable interest in developing advanced methods that enhance phase space sampling and overcome this time scale problem.^6−12^

A popular class of such advanced sampling methods is the so-called collective variable (CV) based enhanced sampling methods. In these methods, we identify a few relevant coarse-grained order parameters, that is, CVs, that correspond to essential slow degrees of freedom. Typically, the selection of CV is made manually by using physical and chemical intuition^13−15^ and sometimes requires a bit of trial and error, while methods based on machine learning are also showing great promise in automating this task.^16−19^ The slow molecular process of interest is then associated with free energy barriers separating metastable states on the free energy surface (FES) as a function of the chosen CVs. We then enhance the sampling of the FES by introducing an external bias potential that is adaptively constructed on the fly during the simulation to reduce or even wholly flatten free energy barriers. We can trace the idea of biased sampling to the original umbrella sampling method introduced in 1977.^20^ The main difference between CV-based enhanced sampling methods lies in how they construct the bias potential and which kind of biased sampling is obtained. Some examples of methods that fall into the category of CV-based enhanced sampling techniques are local elevation,^21^ adaptive biasing force,^22−24^ energy landscape paving,^25^ multiple windows umbrella sampling,^26^ Gaussian-mixture umbrella sampling,^27^ nonequilibrium umbrella sampling,^6,28^ metadynamics,^29−31^ metabasin metadynamics,^32^ parallel-bias metadynamics,^33^ basis function sampling,^34^ Green’s function sampling,^35^ artificial neural network sampling,^36^ reweighted autoencoded variational Bayes for enhanced sampling,^37^ on-the-fly probability-enhanced sampling,^38,39^ adaptive topography of landscapes for accelerated sampling,^40^ and reweighted Jarzynski sampling.^41^

Variationally enhanced sampling (VES)^42^ is a recently developed CV-based enhanced sampling method based on a variational principle. It introduces a convex functional of the bias potential that is related to the relative entropy and the Kullback–Leibler divergence.^43^ To minimize the functional, we generally take the bias potential as a linear expansion in some basis function set. Bias potentials based on a neural network^44^ or free energy models^45−48^ have also been considered in the literature. VES not only allows for obtaining FESs but can also be used to obtain kinetic properties.^49^

The focus of this paper is the choice of basis set in the linear expansion of the bias potential within VES. So far, the basis functions employed have been primarily global functions such as plane waves, Chebyshev, or Legendre polynomials that are orthogonal but delocalized in the CV space. Gaussian basis functions have also been used.^50,51^ However, there has not been an extensive study of how the choice of the basis functions affects the convergence behavior. In particular, it remains an open question if basis functions that are localized in the collective variable space might perform better. While Gaussian basis functions might be the type of localized basis functions that first comes to mind, they have the disadvantage of not forming orthogonal basis sets. Instead, a more appealing option might be Daubechies wavelet-based basis sets,^52^ as they are orthogonal and exhibit an attractive multiresolution property. Daubechies wavelets have recently been used as basis functions for other applications within molecular simulations, such as density functional theory^53,54^ or coarse-grained potentials.^55^

In this work, we introduce the Daubechies wavelets as basis functions for the variationally enhanced sampling method. We implement the wavelets into the PLUMED 2 code,^56^ tune their parameters, and evaluate their performance on various systems, going from model potentials to the calcium carbonate association process in water.^57^ We also test Gaussians and cubic B-splines as other types of localized basis functions. Section 2 presents the theory of the VES method and introduces the new basis functions. Besides the theoretical properties, we also provide details on the implementation of the new functionality into the VES module of PLUMED 2.^56^ In Section 3, we present the computational details of the benchmark systems. We discuss the results of the simulations in Section 4, and in Section 5, we end with some concluding remarks.

## 2 Theory and Methodology

### 2.1 CV-Based Enhanced Sampling

We consider a molecular system described by the set of atomic coordinates  and a potential energy function *U*(). Without the loss of generality, we limit our discussion to the canonical (NVT) ensemble in the following. The Boltzmann distribution, which we want to sample by molecular dynamics (MD) or Monte Carlo simulations, is defined as
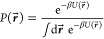
1where β = (*k*_B_*T*)^−1^ is the inverse of the thermal energy. In collective variable (CV) based enhanced sampling methods, we identify a few relevant CVs that correspond to critical slow degrees of freedom. The equilibrium probability distribution corresponding to a set of CVs, ***s***() = {*s*_1_(), *s*_2_(), ..., *s_N_*()}, is given by
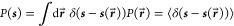
2while the free energy surface (FES) is defined as
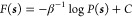
3where *C* is an additive constant.

We are generally interested in systems where the FES (or, equivalently, the equilibrium probability distribution *P*(***s***)) is hard to sample by unbiased molecular dynamics simulations. For example, the FES might be characterized by many metastable basins separated by high free energy barriers such that barrier crossings occur on far greater time scales than we can afford in simulations, that is, they are rare events.

To overcome this time scale or rare event problem, we can enhance the sampling by introducing a bias potential *V*(***s***()) that acts in the space of the CVs. The introduction of this bias potential will lead to a biased (i.e., non-Boltzmann) distribution given by
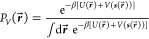
4

Consequently, this leads to a biased CV distribution given by
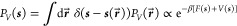
5that is chosen such that the sampling is easier and free energy barriers are reduced or even completely flattened.

From the biased simulation, we can obtain an ensemble average of an observable *O*() for the unbiased simulation through reweighting
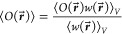
6where *w*() =  is the weight of configuration  and the averages on the right side are obtained in the biased ensemble. In particular, we can obtain the FES for some CV set ***s***′ by using  = δ(***s***′ – ***s***′())
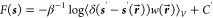
7where we can ignore the denominator in eq 6 as it only gives a constant shift of the FES (i.e., we can include it in the constant *C*^′^). In practice, the reweighted FES is obtained using a reweighted histogram or kernel density estimation where each sample is weighted by the bias acting on it, *w*() = . The reweighting procedure of eq 6 assumes a fixed bias potential, but often, it can be used for adaptively constructed bias potentials under the assumption that the bias potential is quasi-stationary, as we discuss below.

### 2.2 Variationally Enhanced Sampling

In the VES method introduced by Valsson and Parinello,^42^ the bias potential is constructed by minimizing a convex functional given by
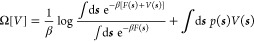
8where *p*(***s***) is a normalized probability distribution. The stationary point of this functional is given up to a constant by
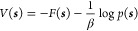
9which, due to the convexity of Ω[*V*], is the global minimum. At this minimum, the CVs are distributed according to *p*(***s***), which is consequently called a “target distribution”. It can be shown that the Ω[*V*] functional is related to the Kullback–Leibler divergence (or relative entropy) and the cross entropy.^43^

Thus, by minimizing Ω[*V*], we can construct a bias potential that leads to a sampling of the CVs according to the target distribution *p*(***s***). The most straightforward choice of the target distribution is a uniform target distribution, leading to completely flat sampling in the CV space. However, we have found it better to employ a so-called well-tempered target distribution^30,58^ given by *p*(***s***) = [*P*(***s***)]^1/γ^/ ∫ d***s*** [*P*(***s***)]^1/γ^, where γ is a parameter, named bias factor, that determines how much the sampling is enhanced as compared to the equilibrium distribution *P*(***s***).

We can determine the FES directly from the bias potential through eq 9. Alternatively, we can obtain the FES, both for the biased CVs and also for any other set of CVs, by using the reweighting procedure shown in eq 6. While the VES bias potential is time-dependent, it quickly becomes quasi-stationary. Therefore, this reweighting procedure is valid after a short initial transient in the time series that is ignored. Note that, differently from metadynamics,^31,59^ we generally do not need to account for time-dependent constants when performing reweighting with VES. Furthermore, under certain conditions, the VES method can also be used to obtain kinetic properties.^49^

In practice, we perform the minimization of the Ω[*V*] functional by assuming a functional form of the bias potential *V*(***s***; **α**) that depends on a set of variational parameters **α** = {α_1_, α_2_, ..., α_*M*_}. Thus, we go from an abstract functional minimization to a minimization of the multidimensional function Ω(**α**).

The most general strategy is to take the bias potential to be a linear expansion in some set of basis functions ***f*** = {*f*_1_, *f*_2_, ..., *f_M_*}:
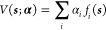
10We can then obtain the gradient ∇Ω(**α**) and the Hessian *H*_Ω_(**α**) as
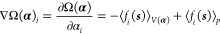
11
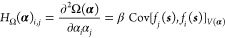
12where angular brackets denote expectation values and Cov[···] is the covariance, obtained either over the bias potential or over the target distribution.

Due to statistical sampling, the estimates of the gradient and Hessian are generally noisy. Therefore, we perform the minimization of Ω(**α**) using stochastic optimization algorithms. In particular, the averaged stochastic gradient descent algorithm from ref (60) has proven to be a convenient choice. In this algorithm, the instantaneous parameters are updated according to the following recursion equation:
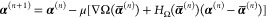
13where μ is a constant step size and the gradient and Hessian are obtained using the averaged parameters  (i.e., the bias potential depends on the averaged parameters). The parameters are updated with a relatively small stride, on the order of 1000 MD steps. Here, we only employ the diagonal part of the Hessian matrix, as generally done in VES.^42,43^

### 2.3 Linear Basis Functions for VES

The focus of this paper is the basis functions used in the linear expansion of the bias potential (eq 10). So far, the basis functions employed have been global functions such as plane waves (i.e., Fourier series),^42^ Chebyshev polynomials,^58^ or Legendre polynomials. The usage of global functions is closely related to the idea of using spectral methods for function approximation.^61^ Favorable for their usage within VES, these basis functions form complete and orthogonal basis sets. However, they are delocalized in the CV space. In other words, they are non-zero over their full domain except on isolated points.

Using global or delocalized basis functions means that, during the optimization process, the bias potential will change even in parts of CV space where the MD simulation is not currently exploring. While this has not proven to be a significant issue, it is clear that delocalized basis functions might not be the optimal choice.

In this work, we consider the performance of using VES with localized basis functions, that is, functions that are non-zero on only some part of the domain of the bias potential. Therefore, they should not suffer from the issue of the bias potential changing in parts of CV space that the simulation is not currently exploring.

Examples of such localized basis functions that come to mind would be Gaussians or splines. In fact, in refs (50) and (51), the authors employed VES with Gaussian basis functions. The results obtained with this VES setup were found to be inferior to some of the results obtained with other enhanced sampling methods used by the authors (such as umbrella sampling^20^), but as no other basis functions were used with VES, it is hard to judge the performance of the Gaussian basis from their results. However, one disadvantage with using Gaussians or splines as basis functions is that they do not form orthogonal basis sets, which might affect the optimization process.

We have thus been motivated to explore the usage of wavelets as basis functions. In particular, we consider Daubechies wavelets,^52,62^ which are localized functions that form orthogonal and complete basis sets. Furthermore, they have an intrinsic multiresolution property that makes it possible to iteratively add more basis functions on smaller scales in a way that preserves the orthogonality of the basis.

**Figure 1:**
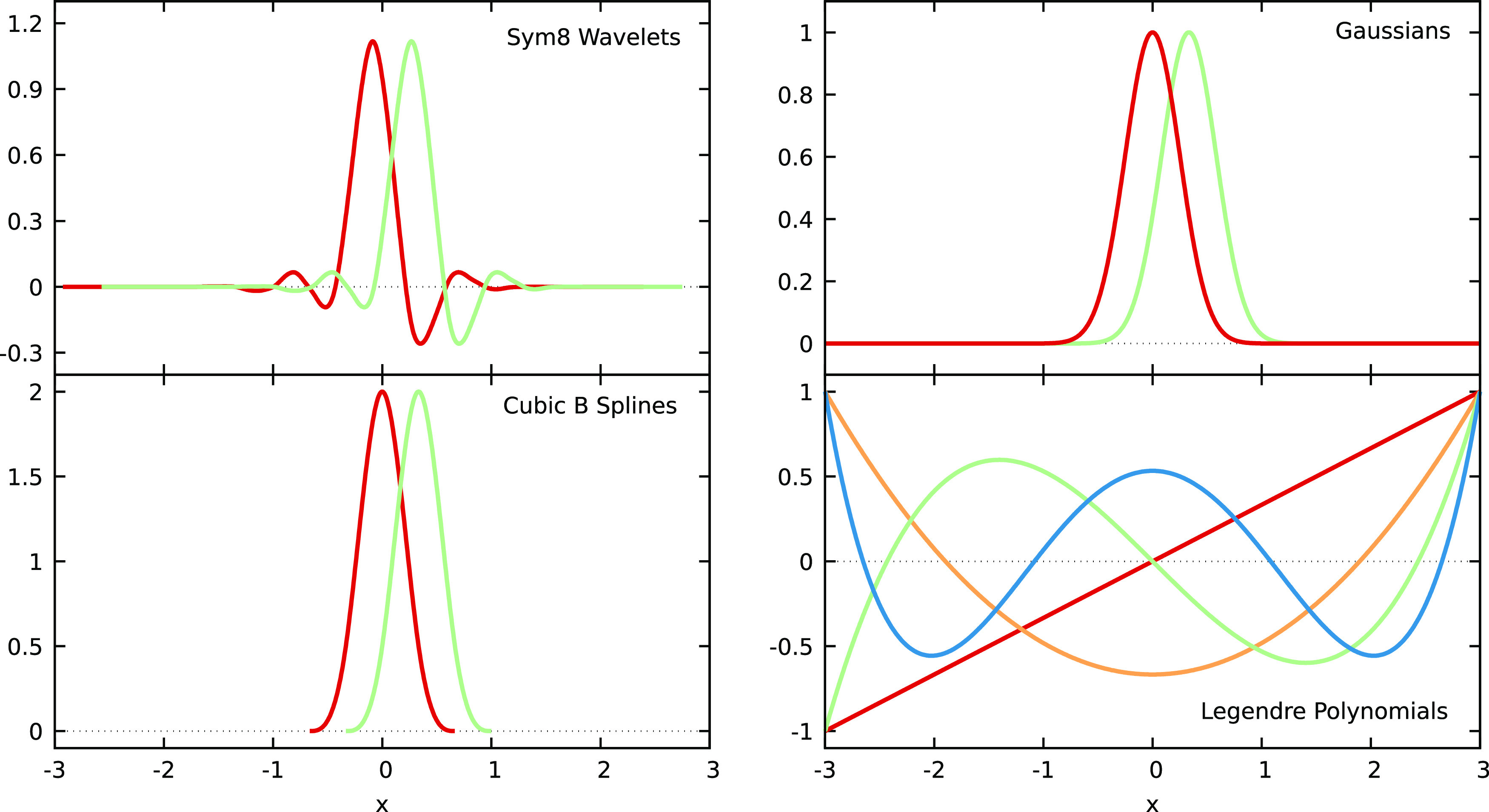
Visualization of different VES basis functions used in this paper. The Sym8 wavelets, Gaussians, and cubic B-splines are localized basis functions. Here, we only show two adjacent functions, while a full basis set would include all shifted functions in the given interval (that is, [−3, 3] here). On the contrary, Legendre polynomials are delocalized functions supported on the full interval of the bias. The Legendre basis set consists of all polynomials up to a certain order; the figure shows the functions up to the quartic polynomial.

In the following sections, we briefly describe the new localized basis functions—Daubechies wavelets, Gaussians, and cubic B-splines—as well as Legendre and Chebyshev polynomials that we consider for comparison. These basis functions are shown in Figure 1. We give descriptions of one-dimensional basis functions only, as basis sets for higher dimensions can be obtained by considering a tensor product. For example, in two dimensions, we obtain
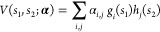
14where *g_i_*(*s*_1_) and *h_j_*(*s*_2_) are some one-dimensional basis functions. All the one-dimensional basis sets described in the following are defined on some given interval [*a*, *b*] and include an additional constant basis function. In practice, for MD simulations, we also need the derivatives of the basis functions to obtain the biasing force due to the external bias potential, but this is a straightforward task for all of the basis functions considered here.

### 2.4 Daubechies Wavelet Basis Functions

Daubechies developed a theory for special types of wavelets that can be used to construct complete and orthogonal basis functions.^52^ These wavelets are based on using a pair of functions: the scaling function (or father wavelet) ϕ and the wavelet function (or mother wavelet) ψ. They are defined by
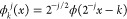
15
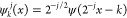
16for a given scale  and shift . The exact properties are set by choosing the filter coefficients *h_k_* and *g_k_* in the refinement relations given by
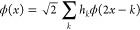
17
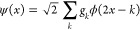
18

Daubechies proved that certain finite sets of filter coefficients result in orthonormal bases. Using these wavelet functions, any square-integrable function *g(x)* can be approximated up to an arbitrary precision by a linear combination with coefficients **α**
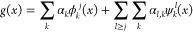
19where the wavelet functions satisfy orthogonality relations:^63^
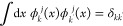
20
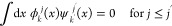
21
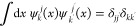
22

We can see the multiresolution property of the wavelet basis functions in eq 19. Starting with the father wavelets ϕ at some scale *j*, an increasingly more accurate approximation is obtained by adding mother wavelets ψ at finer scales.

In this paper, we will focus on the coarsest approximation only, which corresponds to a single level of father wavelets at some scale *j*
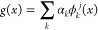
23The exact wavelet type and the scale are left for us to choose.

The wavelet type is determined by the set of filter coefficients *h_k_* and *g_k_*. Desirable properties for our application are small support of the individual function, at least *C*^1^ regularity (one continuous derivative), and the reproduction of polynomials up to a desired order.

The wavelets developed by Daubechies satisfy these properties and in fact result in the minimally supported functions for a given polynomial order. In this paper, we consider filter coefficients that result in the least asymmetric variant of these wavelets or so-called symlets.^52^ The reduced asymmetry of the symlets comes at the cost of slightly reduced regularity as compared to the conventional maximum phase Daubechies wavelets. However, this does not cause problems as we only require one continuous derivative. In practice, we found the symlets to perform better than the maximum phase Daubechies wavelets. The symlets are also used in wavelet-based density functional theory calculations.^54^ We will denote the symlets by Sym*N*, where *N* is equal to half the number of coefficients used for construction.

The chosen number *N* determines the properties of the symlets, including the number of vanishing moments of the mother wavelet. Having *N* vanishing moments means that all polynomial functions up to the order *N* – 1 are orthogonal to the mother wavelet. Consequently, any polynomial of order up to *N* − 1 can be represented exactly by a single level of the father wavelet ϕ (i.e., the scaling function). Employing a wavelet basis with a larger *N* can thus helps to construct a bias potential with less regularity and steeper slopes. On the other hand, the range over which the wavelet functions are non-zero is proportional to 2*N* – 1. Because the basis consists of integer-shifted functions, a larger support (i.e., non-zero range) results in more overlap between functions. This makes it necessary to use more basis functions at the same scale and thus results in more expansion coefficients to optimize. After some testing, we found that using Sym8 or Sym10 yields the best results for the systems considered in this paper. Further discussion and a comparison of symlets with different numbers of vanishing moments can be found in Section S1 of the Supporting Information (SI).

The scale *j* of the wavelet basis can be chosen freely. Instead of selecting the scale directly, we set the desired number of basis functions. In principle, there is an infinite number of shifted wavelet functions in the basis. However, only a few of them are supported inside the range [*a*, *b*] on which the bias potential is defined. Furthermore, they are non-zero only on a small part of their domain. Thus, we choose to only include the ones with any (absolute) function value inside the bias range that is at least 1% of the maximal function value. We then calculate the required scaling to arrive at the desired number of basis functions. We did not observe disadvantages from excluding wavelets with minor contributions, while it allows us to reduce the number of coefficients to be optimized.

Generally, using a smaller scale and, consequently, more basis functions allows us to represent finer features better at the cost of needing to optimize more variational parameters. In Section S1 of the SI, we show results where we change the number of the basis functions for a fixed *N* value.

### 2.5 Gaussian Basis Functions

Gaussian basis functions are given by the mathematical expression
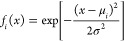
24where μ_*i*_ is the center of the individual Gaussian and σ is a constant width parameter. The full basis set is then given by Gaussian functions with centers distributed evenly on the interval [*a*, *b*]. We add the first center at μ_0_ = *a* and define the shift between centers as *d* = μ_*i*_ – μ_*i* – 1_ = (*b* – *a*)/*N*, where *N* is a user-specified integer fixing the number of basis functions.

To mitigate systematic errors at the boundaries, we add one function on each side outside the range, resulting in a total of *N* + 3 basis functions including the constant. As the force from the VES bias is zero outside the chosen interval by design, these additional functions will only contribute inside the bias range, similar to the boundary correction approach for metadynamics in ref (64). Although more complicated boundary correction algorithms have been developed,^65,66^ we found our simple approach to work well.

The width σ of the Gaussians is set by the user. For a fixed number of Gaussians, the possible resolution of the basis can be increased by choosing Gaussians with a smaller width. However, reducing the width will reduce the overlap between Gaussians, and a too-small width will result in an ill-behaving basis set. Thus, the optimal width, which very likely is system dependent, is the smallest one that still results in good convergence. In refs (50) and (51), the width σ was set to be equal to the distance *d* between the centers of the Gaussians. However, as shown in Section S2 in the SI, we found improved performance when using a smaller width of σ = 0.75*d*. Because this yielded better results for the model systems considered here, we will show only Gaussian results obtained with this optimal width in the rest of the paper, while we refer the reader to the SI for results obtained with other σ values.

### 2.6 Cubic B-Spline Basis Functions

We consider the cubic B-spline basis functions from ref (67) that are given by the mathematical expression
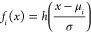
25where
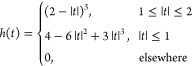
26Here μ_*i*_ is the center of the cubic B-spline basis function, and σ is the width. The full basis set is then given by spline functions with centers distributed evenly on the interval [*a*, *b*]. The first center is set on the left boundary μ_0_ = *a*, and we define the shift between centers as *d* = μ_*i*_ – μ_*i* – 1_ = (*b* – *a*)/*N*, where *N* is a user-specified integer fixing the number of basis functions. Similar to the Gaussian basis functions, to avoid boundary effects, we add functions on each side outside the range, resulting in a total of *N* + 3 basis functions including the constant. Different from the Gaussians, the width σ is fixed and taken as equal to the distance between centers, σ = *d*.

### 2.7 Legendre and Chebyshev Polynomial Basis Functions

Legendre and Chebyshev polynomials form sets of orthogonal basis functions on a closed interval that is matched to the range of the bias potential. Contrary to the previously described bases, the basis functions are not localized in a specific part of the interval but are non-zero except on isolated points. Chebyshev polynomials of the first kind are given by the recursion relations
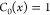
27
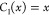
28
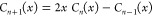
29while the recursion relations of the Legendre polynomials are
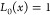
30
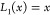
31
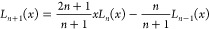
32

Both Chebyshev and Legendre polynomials are defined intrinsically on the interval [−1, 1] and need to be scaled and shifted when employed on different intervals. For a given interval [*a*, *b*], we use the following function to transform *t* ∈ [*a*, *b*] to *x* ∈ [−1, 1]:
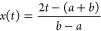
33

### 2.8 Implementation of New Basis Functions

We have implemented the new basis functions into the VES module of the PLUMED 2 code.^56,68^ Our implementation is publicly available in the official PLUMED 2 GitHub repository, and it is released in version 2.8 of PLUMED.

While it was straightforward to implement Gaussians and splines, wavelets pose the problem of not having an analytic mathematical expression. Instead, in the beginning of the simulation, we generate the wavelet values and derivatives on a grid through an iterative scheme. We then use the grid as a lookup table during the simulation. This means that the computational overhead of using the wavelets is minimal. To generate the wavelet grid, both for the values and for the derivatives, we employ a vector cascade algorithm^69^ that relies on finding eigenvectors of a characteristic matrix and subsequent vector–matrix multiplications to iteratively get values on an increasingly finer spaced grid. We calculate the exact values on a grid of at least 1000 points and use linear interpolation to obtain in-between values.

As localized functions are non-zero only in a small region of the total CV space, we have to modify the optimization scheme slightly. If there is no sampling in the non-zero region of a basis function during one iteration of the bias potential, the elements of gradient and Hessian corresponding to that basis function are set to zero before updating the variational parameters. This is needed because the gradient elements for these basis functions might still be non-zero due to the average over the target distribution (the second term in eq 11). Setting them to zero prevents erroneous updates of variational parameters if no sampling of the non-zero region occurred. Note that this procedure is done only for individual elements, so the total gradient vector and Hessian matrix still include non-zero elements.

We note that our implementation of the wavelet, Gaussian, and spline basis functions also supports periodic CVs. Furthermore, in addition to the least asymmetric wavelets (i.e., symlets) that we use in this work, the wavelet implementation also supports conventional maximum phase Daubechies wavelets. However, we found the latter to perform worse when compared to the symlets.

## 3 Computational Details

To evaluate the performance of the different basis functions, we perform simulations on different systems, going from model potentials in one and two dimensions to a realistic system modeling the association process of calcium with carbonate in water.

### 3.1 Double-Well Potential

**Figure 2:**
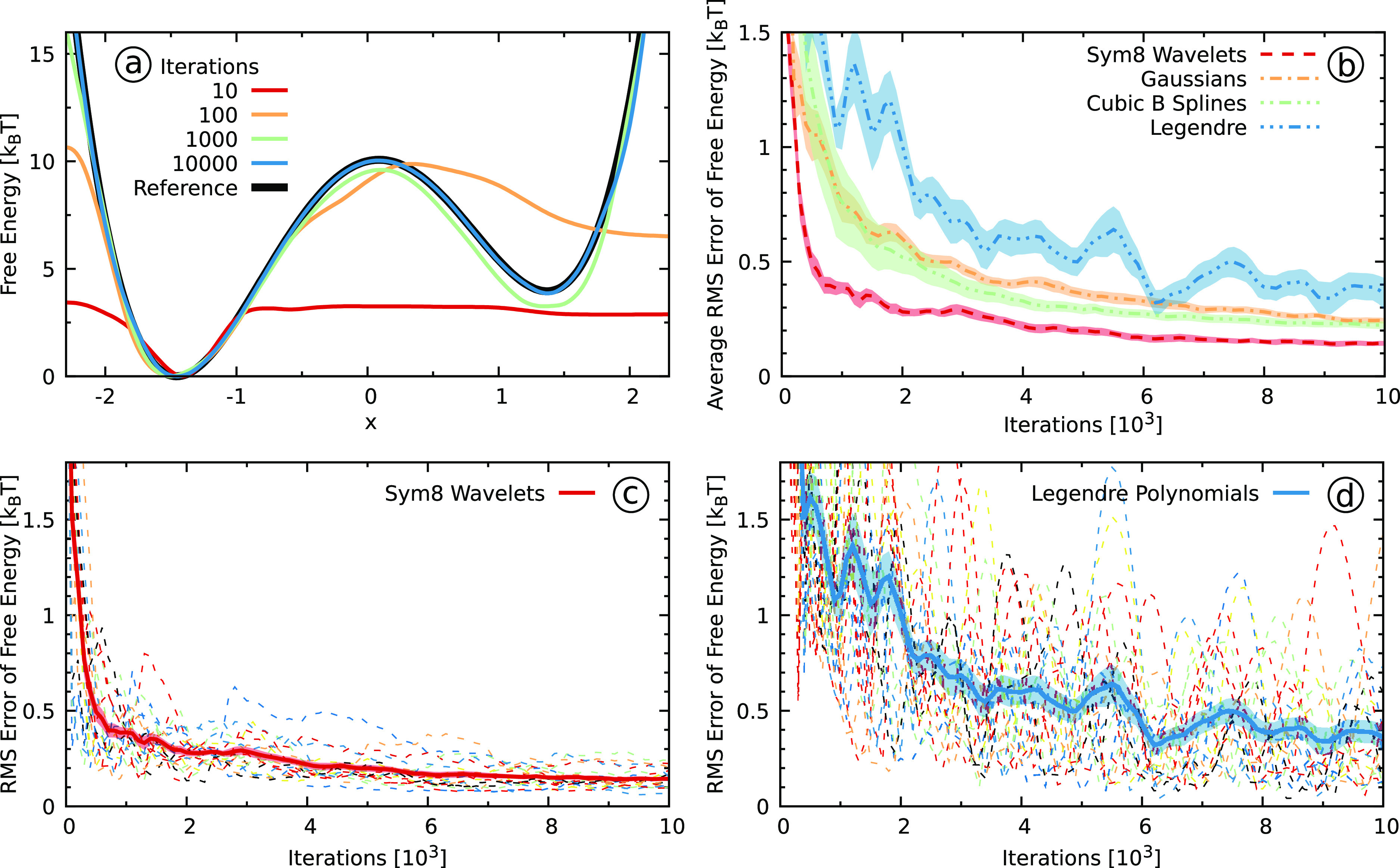
Results for the one-dimensional double-well potential described in Section 3.1. (a) The reference FES along with the FES obtained using the wavelet basis functions at different numbers of bias iterations for one of the runs. (b) The RMS error measure (Section 3.5, eq 36) for the different basis functions as a function of the number of bias iterations. The lines denote the average over 20 independent runs, and the shaded areas denote the corresponding standard error. (c, d) The RMS error of the individual runs for (c) Sym8 wavelets and (d) Legendre polynomials. The thick lines are the same as in panel b, and the dashed lines each resemble one of the runs.

We start by considering a single particle moving in a one-dimensional model potential given by
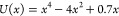
34that has two states separated by a barrier of around 5 energy units. The form of this potential can be seen in Figure 2a. We take the *x*-coordinate as the CV such that the reference FES will be given by the potential above, *F*(*x*) = *U*(*x*) (up to an additive constant). We employ the ves_md_linearexpansion command line tool from the VES code for the simulations. The ves_md_linearexpansion tool implements a simple molecular dynamics integrator with a Langevin thermostat.^70^ We use a time step of 0.005 and a friction coefficient of 10 for the Langevin thermostat. We set the temperature to *T* = 0.5/*k*_B_ such that the barrier height is about 10 *k*_B_*T* (*k*_B_ = 1). We choose to run simulations with four different basis sets: Sym8 wavelets, Gaussians, cubic B-splines, and Legendre polynomials. We expand the bias potential in the interval from −3 to 3 and fix the number of basis functions to 22 for each basis set to allow for a fair comparison. We employ a uniform target distribution and update the coefficients of the bias potential every 500 steps. The step size μ in the averaged stochastic gradient descent optimization algorithm (eq 13) was adjusted to yield the fastest convergence for each basis set. We set it to μ = 0.5 for simulations using localized basis functions and decrease it to μ = 0.1 for the simulations with Legendre polynomials. Each simulation is run for 5 × 10^6^ steps, while the FES was determined every 5 × 10^4^ steps via eq 9. For each basis set, we run 20 independent simulations that are started in the global minimum with different random seeds for the initial velocities and random forces.

### 3.2 Wolfe–Quapp Potential

**Figure 3:**
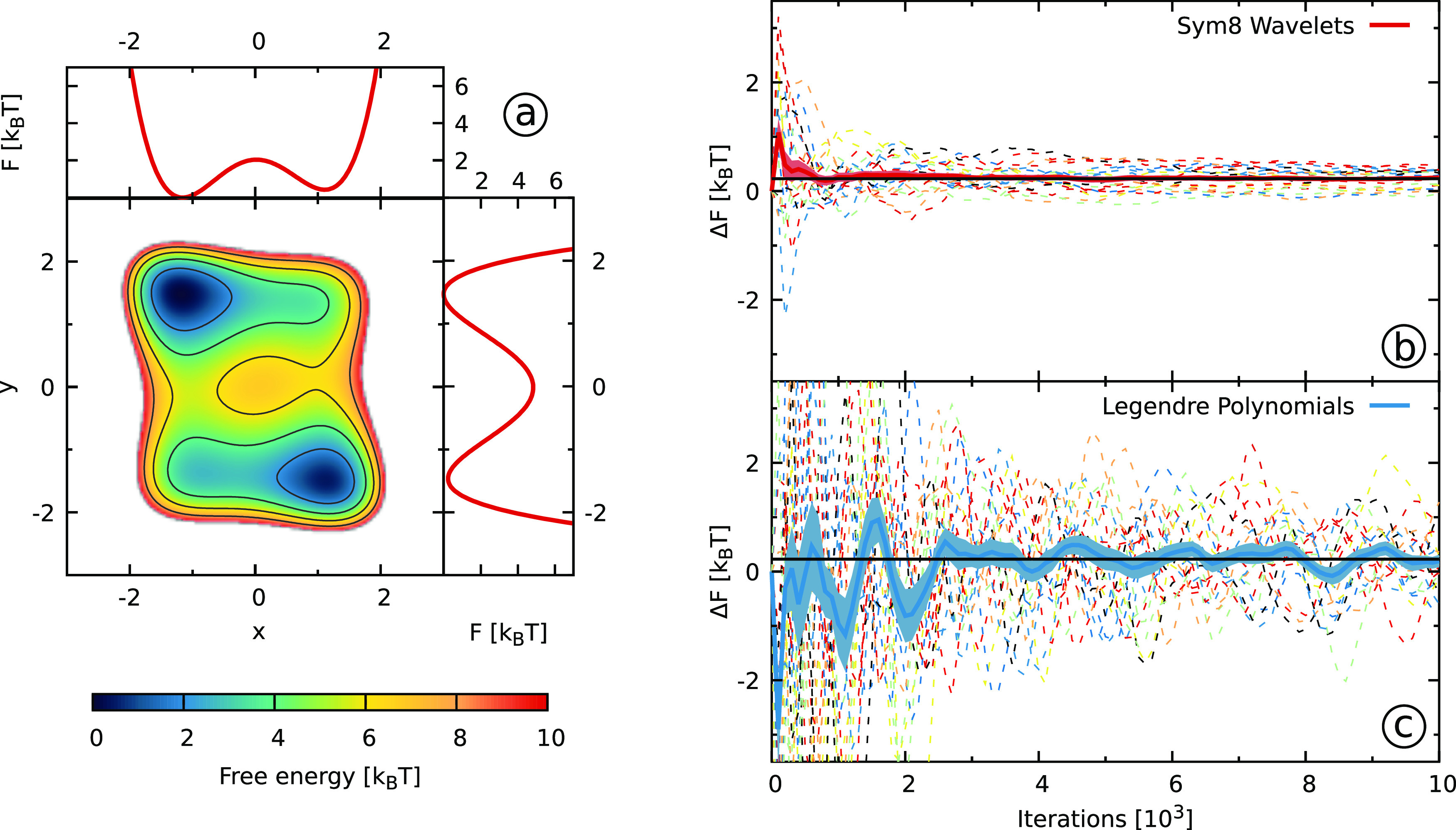
Results for the two-dimensional Wolfe–Quapp potential described in Section 3.2. (a) The reference FES along with free energy projections on the *x*- and *y*-coordinates. (b, c) The free energy difference Δ*F* (Section 3.5, eq 38) between the two states obtained using (b) Sym8 wavelets and (c) Legendre polynomials as a function of the number of bias iterations. We show results from 20 independent simulations with dashed lines. We use solid lines for the averages and shaded areas to denote the standard errors. We denote the reference value with solid black lines. To define the areas corresponding to the two different states, we use the *y* = 0 line.

The second model potential is the two-dimensional Wolfe–Quapp potential:^71,72^
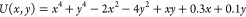
35that has two states separated by a high barrier along the *y*-coordinate, while along the *x*-coordinate, the mobility is high. The potential can be seen in Figure 3 along with projections on the *x*- and *y*-coordinates. We take both the *x*-coordinate and the *y*-coordinate as CVs such that the reference FES will be given by the potential *F*(*x*, *y*) = *U*(*x*, *y*) (up to an additive constant). We bias both CVs in the interval from −3 to 3 using 22 basis functions per CV (484 two-dimensional basis functions in total). We set the temperature to *T* = 1/*k*_B_. We set the step size for all simulations to μ = 0.5. We run 20 independent simulations for each basis set. Otherwise, we employ the same basis functions and simulation parameters as for the one-dimensional potential in the previous section.

### 3.3 Rotated Wolfe–Quapp Potential

**Figure 4:**
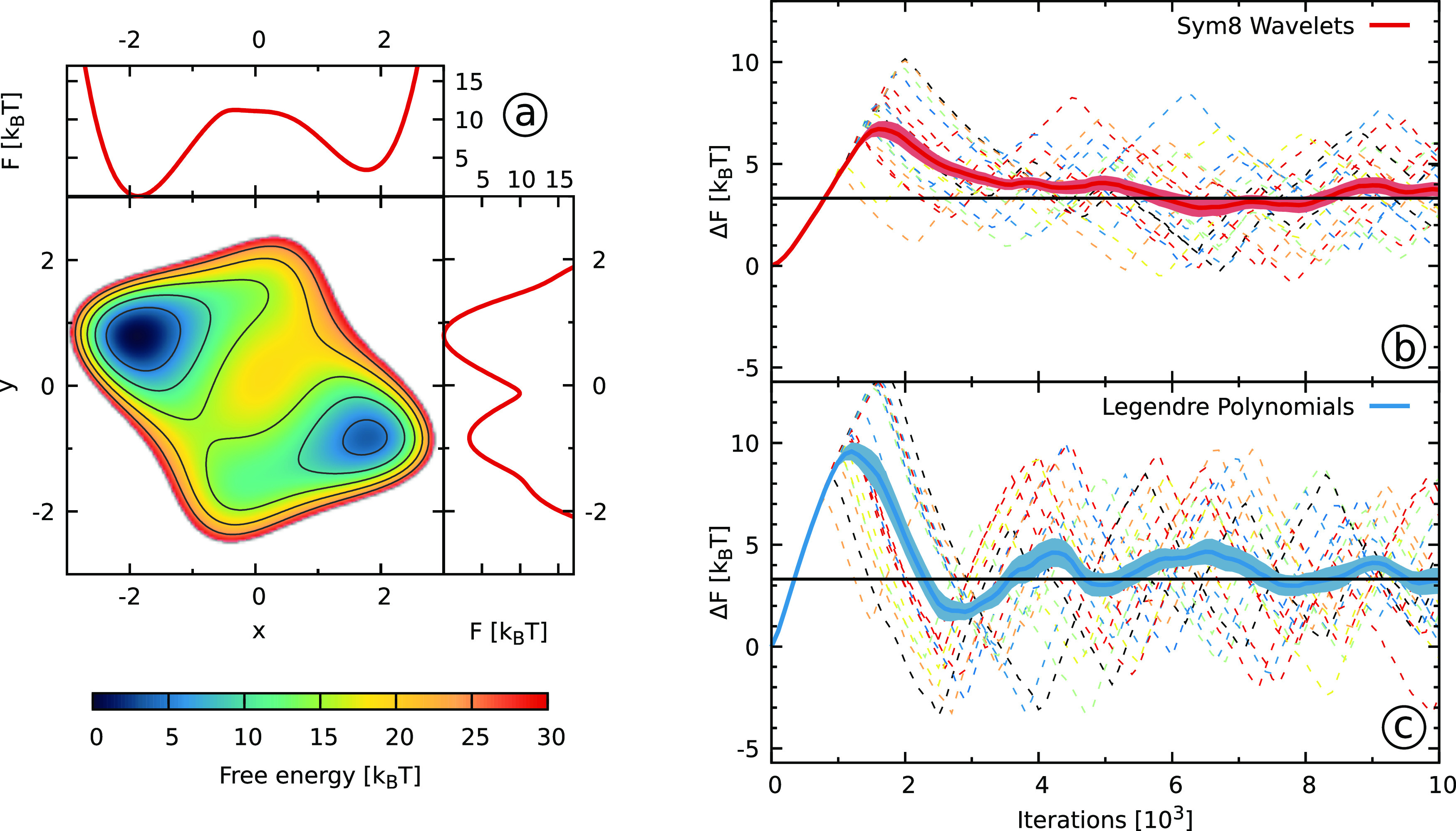
Results for the rotated two-dimensional Wolfe–Quapp potential described in Section 3.3. (a) The reference FES along with free energy projections on the *x*- and *y*-coordinates. Only the *x*-coordinate is biased. (b, c) The free energy difference Δ*F* (Section 3.5, eq 38) between the two states obtained using (b) Sym8 wavelets and (c) Legendre polynomials as a function of the number of bias iterations. We show results from 20 independent simulations with dashed lines. We use solid lines for the averages and shaded areas to denote the standard errors. We denote the reference value with solid black lines. To define the areas corresponding to the two different states, we use the *x* = 0 line.

To test the behavior when biasing only a suboptimal CV, we consider a rotated and scaled version of the Wolfe–Quapp potential. As in ref (48), the potential is rotated by an angle of θ = −0.15π. The potential energy surface is given in Figure 4 together with projections on the *x*- and *y*-coordinates. We take only the *x*-coordinate as a biased CV, which results in missing orthogonal slow degrees of freedom (the *y*-coordinate). The reference FES for the *x*-coordinate can be obtained by integrating over the *y*-coordinate, *F*(*x*) = −β^–1^ log ∫ d*y* e^–β*U*(*x*, *y*)^. We use a temperature of *T* = 1/*k*_B_. We expand the bias potential in the interval from −3 to 3 and fix the number of basis functions to 22 for each basis set. We employ a uniform target distribution and update the coefficients of the bias potential every 500 steps. Otherwise, we employ the same basis functions and simulation parameters as for the previous two model potentials.

For this system, we observe that using the averaged stochastic gradient descent optimization algorithm does not yield good convergence for the localized basis functions. Therefore, we use the Adam stochastic gradient descent algorithm,^73^ which has been used previously for VES in combination with neural networks.^44^ Details of the Adam algorithm can be found in Section S3 in the SI. We notice a high sensitivity of the convergence to the step size η of the Adam algorithm. Although the standard value of η = 0.001 works in most cases, the convergence of the bias is slow, especially for simulations with Sym8 wavelets. Increasing it to η = 0.005 provides much better behavior, whereas increasing it even further results in nonconverging simulations with Legendre polynomials. We use η = 0.005 for all simulations with the Adam algorithm but note explicitly that the choice of parameters seems crucial for good convergence.

While the usage of the Adam algorithm helps improve the convergence for this system, we find a worse performance in comparison to the averaged stochastic gradient descent algorithm when testing it on the other systems considered in this paper. Therefore, further investigation is needed to understand the optimal choice for stochastic optimization. The choice very likely depends on the form of the bias potential (e.g., a linear expansion versus a neural network^44^ or a bespoke model^45−48^) and the basis functions used. An interesting idea might be to combine ideas from different algorithms, similar to what was done in ref (48) where the authors introduced a combination between AdaGrad and Bach’s algorithms. However, a detailed investigation of the stochastic optimization algorithm used within VES is beyond the scope of the current work.

### 3.4 Calcium Carbonate Association

To study the performance of wavelet basis functions for a realistic system, we consider the association process of a calcium carbonate ion-pair in water. We use the LAMMPS code^74^ (5 June 2019 release) interfaced with the PLUMED 2 code for the simulations. We employ the calcium carbonate force field developed in refs (75) and (76) and the SPC/Fw^77^ water model. We follow the computational setup used in a previous metadynamics study of the association process^57^ using this force field. We set up a system that contains a single Ca^2+^–CO_3_^2–^ ion-pair and 2448 water molecules in a periodic cubic box. We equilibrate the system in the NPT ensemble at a constant temperature of 300 K and a constant pressure of 1 bar for 500 ps. All subsequent simulations are performed in the NVT ensemble using a constant temperature of 300 K and a cubic box with side lengths of 41.69 Å. We run 5 ns of unbiased MD simulations from which we select in total 75 snapshots that we use as initial configurations for the biased simulations. We employ a time step of 0.001 ps. All simulations are performed at a constant temperature of 300 K using a Nosé–Hoover thermostat^78−80^ with a chain length of 5 and a relaxation time of 0.1 ps. For the NPT equilibration, we employ a Nosé–Hoover barostat with a relaxation time of 1 ps to keep a constant pressure of 1 bar. Electrostatic interactions are calculated according to the PPPM method^81^ with an accuracy of 10^–5^.

We use the same CVs as in ref (57), namely, the distance between the Ca and C atoms and the coordination number of Ca with water (see Section S5 in the SI for further details). As in the original work,^57^ we use the technique of multiple walkers^82^ with 25 walkers running in parallel to improve convergence, where each walker starts from a different initial configuration. We employ Sym10 wavelets or Chebyshev polynomials as basis functions. For the CV corresponding to the distance between the Ca ion and C atom of the carbonate ion, we use 60 basis functions in the range from 2 to 12 Å. For the CV corresponding to the coordination number, we use 30 basis functions in the range 5 to 9. The total number of two-dimensional basis functions is then 1200. Due to the usage of multiple walkers, we update the coefficients of the bias potential more frequently, that is, every 10 MD steps (the total number of data points for each iteration is then 250). We use the averaged stochastic gradient descent optimization algorithm with a step size of μ = 0.001 for the Sym10 wavelets. For simulations with Chebyshev polynomials, this does not always result in stable simulations, and we use a lower step size of μ = 0.0005 for these. We employ a well-tempered target distribution^58^ with a bias factor of 5, where the target distribution is iteratively updated every 100 bias potential updates (1000 MD steps). We run each walkers for 3 ns, resulting in a cumulative simulation time of 75 ns.

For comparison, we also perform a well-tempered metadynamics (WTMetad)^30^ simulation using the same setup as in ref (57). The bias factor is set to 5. For the Gaussians, we use an initial height of 1 *k*_B_*T* and widths of 0.2 Å and 0.1 for the distance and coordination number, respectively. We deposit Gaussians every 1 ps (1000 MD steps). For the metadynamics simulations, we also run each walker for 3 ns, resulting in a cumulative simulation time of 75 ns.

To focus the sampling in the part of the configuration space of interest for the association process, we add an artificial repulsive wall at a Ca–C distance of 11 Å in all simulations to prevent the ions from moving further apart. In practice, this is implemented by a harmonic bias of the form κ(*x* – *x*_0_)^2^ where we set the parameters to κ = 12 eV and *x*_0_ = 11 Å.

To obtain the reweighted FESs, we employ a reweighted kernel density estimation as implemented in PLUMED 2. We use Gaussian kernels with bandwidths of 0.05 Å and 0.05 for the Ca–C distance and coordination number CV, respectively. We ignore the first 200 ps of each walker and use samples obtained every 0.1 ps. For the metadynamics simulations, we use the *c(t)* reweighting scheme described in refs (31) and (59). During the metadynamics simulations, we calculate the time-dependent constant *c(t)* needed for the biasing weights every time a Gaussian is added using a grid of 275 × 300 over the domain [2,13] × [3,10].

To assess the stability of the simulations, we perform three independent runs using different initial configurations for each of the three biasing setups (VES with wavelets, VES with Chebyshev polynomials, and WTMetaD).

### 3.5 Performance Measures

To evaluate and compare the performance of the basis functions, we consider two different performance measures: the root mean square error with respect to a reference and the free energy difference between some two metastable states.

To measure the quality of the FES *F*(***s***) obtained directly from the bias through eq 9, we calculate the root mean square (RMS) error of the FES with respect to a reference as done in refs (58) and (83). Given some reference FES *F*_ref_(***s***), the RMS error is given by
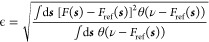
36where we perform the integration over the full CV space and θ is a Heaviside step function such that only regions with a free energy lower than a threshold value ν are considered. Since the FESs are only determined up to a constant, we shift them by their average value in the region of interest, that is, we use
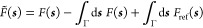
37to calculate the error metric in eq 36, where Γ is taken as the region of CV space where *F*_ref_(***s***) ≤ 4 *k*_B_*T*. We set the parameter ν = 8 *k*_B_*T*. We consider always an ensemble of multiple independent runs that are initiated with different initial conditions because a single simulation might not be representive.^84,85^ We then compare the mean RMS error as well as the associated standard error of the mean.

Another performance measure we can employ is to calculate the free energy difference Δ*F*_*A*, *B*_ between two different states:^31^
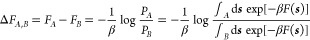
38where the domains of integration are the regions in CV space associated with the states *A* and *B*, respectively.

### 3.6 Data Availability

The data supporting the results reported in this paper are openly available at Zenodo^86^ (DOI: 10.5281/zenodo.5851773). All LAMMPS and PLUMED 2 input files and analysis scripts required to reproduce the results reported in this paper are available on PLUMED-NEST (www.plumed-nest.org), the public repository of the PLUMED consortium,^68^ as plumID:22.001 at https://www.plumed-nest.org/eggs/22/001.

## 4 Results and Discussion

### 4.1 Model Potentials

A common way to test the performance of methodological developments of enhanced sampling methods is to consider the dynamics of a single particle on model potentials that emulate prototypical free energy landscapes. We, therefore, start by considering three model potentials, where we compare the performance of the localized basis functions (Sym8 wavelets, Gaussians, and cubic B-splines) to the delocalized Legendre polynomials that have been used as basis functions within VES so far. For these simulations, we always perform 20 independent runs for each set of basis functions and use the performance measures that we have described in Section 3.5 to compare the FESs obtained from the bias potential via eq 9.

We start by considering the one-dimensional double-well potential shown in Figure 2a that has a high free energy barrier of around 10 *k*_B_*T* when going from the left to right side. In panel a of Figure 2, we show an example of the FES obtained using wavelet basis functions at different bias iterations. In the SI, we present a movie showing the time evolution of the FES of exemplary simulations for all different basis sets. In panel b, we show the RMS error metric (eq 36) for the different basis functions. We can observe that, on average, the FES (or equivalently the bias) converges considerably faster with the localized basis functions than with the delocalized Legendre polynomials. Furthermore, the localized basis functions converge to a better estimate of the FES as indicated by the smaller RMS error. We can observe that the wavelets perform the best of the three localized basis functions.

In Figure 2b, we can also observe considerably larger fluctuations in the average RMS error and a larger standard error for the Legendre polynomials. The reason for this is twofold, as we can see from looking at the RMS error for the individual runs, shown in panels c and d for the wavelets and the Legendre polynomials, respectively. First, within each individual simulation, the bias potential is fluctuating more for the Legendre polynomials. Second, there is a more significant difference between runs for the Legendre polynomials. In comparison, the wavelets show a much more robust behavior with considerably smaller fluctuations within individual runs and more minor differences between runs. We can see a similar effect for the Gaussians and cubic B-splines, although they do not behave as well as the wavelets (see Figure S5 in the SI). Therefore, for this simple system, we can already see the benefits of using localized basis functions.

In the following, we will focus on the wavelets and the Legendre polynomials, while we refer the reader to the SI for results for the Gaussians and cubic B-splines. Furthermore, we will only use the free energy difference to compare the basis functions while presenting the results for the RMS error metric in the SI.

The next system that we consider is the two-dimensional Wolfe–Quapp potential^71,72^ that is a commonly used model potential for testing methods.^72,87−89^ We show its free energy surface, along with the free energy projections on the *x*- and *y*-coordinates, in Figure 3a. The potential has two states separated by a barrier along the *y*-coordinate, while the system is relatively mobile along the *x*-coordinate. Still, due to a strong coupling between the *x*- and *y*-coordinate, it is essential to consider both coordinates as biased CVs to get a good sampling. We thus expand the two-dimensional bias potential in a tensor product basis set of one-dimensional basis functions.

In panels b and c of Figure 3, we show the free energy difference between the two states for the wavelets and the Legendre polynomials, respectively. We can see a rather similar behavior as for the one-dimensional model potential. The wavelets exhibit far smaller fluctuations within individual runs and considerably smaller differences between runs than the Legendre polynomials. Looking at the averaged free energy difference, we can see that the wavelet simulations converge substantially better and faster than the Legendre polynomials. We can draw similar conclusions by considering the RMS error measure shown in Figure S7 in the SI.

We show the estimates of the free energy difference from the simulations with Gaussians and cubic B-spline basis functions in Figure S7 in the SI. We can observe that the Gaussians perform better than the Legendre polynomials but worse than the wavelets. However, we find that cubic B-splines perform the worst of all the basis functions and do not yield usable results for this system.

Finally, we consider a rotated version of the Wolfe–Quapp potential shown in Figure 4a that has been used as a test case for biasing suboptimal CVs.^44,48,90^ We only take the *x*-coordinate as a CV for biasing, so we are missing the *y*-coordinate that is an orthogonal slow degree of freedom. We show the free energy difference between the two states in panels b and c of Figure 4. As expected, due to the usage of a suboptimal CV, the convergence behavior is slightly worse than for the previous two systems, and we need longer simulation times to obtain adequate convergence. Nevertheless, the wavelets exhibit good convergence behavior that, as before, is more robust than for the Legendre polynomials. As shown in Figure S8 in the SI, the Gaussians and the cubic B-splines perform worse than both wavelets and Legendre polynomials.

As discussed in Section 3.3, for this system, we have used a different optimization algorithm, the Adam optimizater,^73^ instead of the averaged stochastic gradient descent.^60^ This choice might explain a slightly different behavior in the time evolution of individual runs as compared to the previous two systems.

Having tested the localized basis functions on three different model systems, we can draw certain conclusions. The wavelet basis functions exhibit much more robust convergence behavior than the Legendre polynomials. For the wavelets, the fluctuations of the bias potential within individual runs are smaller. Additionally, the difference between independent runs is considerably smaller. The Gaussian and the cubic B-spline basis functions perform worse than the wavelets for all considered systems and do not yield usable results for some systems. Therefore, we recommend against their usage. Having established the excellent performance of the wavelets in model systems, we now move on to their use in a more realistic system.

### 4.2 Calcium Carbonate Association

For a more realistic system, we consider the association process of calcium carbonate in water that has previously been investigated in ref (57) using metadynamics simulations. In that work, the authors used the technique of multiple walkers^82^ with 25 walkers to improve the convergence. Here, we will follow the same procedure for the wavelet and Chebyshev polynomial simulations. For comparison, we also run well-tempered metadynamics simulations using the same computational setup as used in ref (57). For each of the three biasing setups (VES with Sym10 wavelets, VES with Chebyshev polynomials, and WTMetaD), we run three independent simulations.

**Figure 5:**
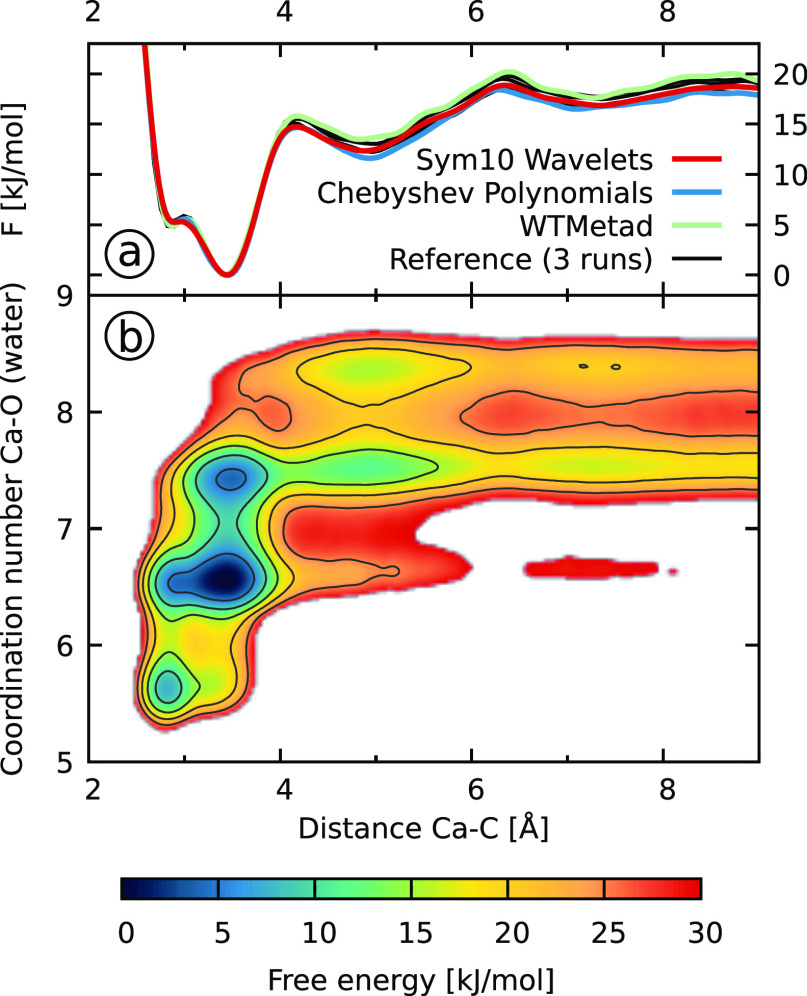
Free energy surfaces for the calcium carbonate system described in Section 3.4. (a) Projections on the distance CV for the FESs obtained directly from the bias via eq 9 (VES) or by summing over the deposited Gaussians (WTMetad). We only show one of the runs for each biasing setup. The reference data are obtained from ref (57). (b) FES as a function of both biased CVs obtained by reweighting one of the wavelet simulations.

In Figure 5b, we show the free energy surface as a function of the two biased CVs: the distance between the calcium and the carbon atom of the carbonate and the coordination number of the calcium to the oxygens of the water molecules. We can see that to fully understand the association process, it is necessary to consider both CVs as the solvation state of the calcium, as measured by the coordination number CV, is closely coupled to the calcium–carbon distance. The minima of the FES with a Ca–C distance smaller than 4 Å correspond to the states with a contact ion-pair. The lowest state of the FES is the monodentate associated state at around 3.5 Å. At a lower coordination number and smaller distance, a second minimum corresponding to the bidentate state can be seen. For larger Ca–C distance, the ions are no longer in direct contact but are separated by the solvent. The states with a distance of around 5 Å correspond to the solvent-shared ion-pair, while the states around 7 Å denote where the solvation shells of the two ions barely touch. For even larger distances, the two ions are fully solvated.

To compare the different simulations, we look at the projections of the FES on the distance CV that is shown in Figure 5a. These free energy profiles are obtained at the end of simulations directly from the bias potential, that is, via eq 9 for the VES simulations or by summing up the deposited Gaussians for the WTMetad simulations. For each of the three biasing setups, we only show one representative free energy profile, while the profiles for the other runs are shown in Figure S9 in the SI. We also show three reference profiles from ref (57). All the free energy profiles are aligned such that their minimum is at zero.

We can observe in Figure 5a that all the free energy profiles obtained from our simulations are in a decent agreement with each other and the reference results from ref (57). All the simulations capture reasonably well the small barrier between the mono- and bidentate states at about 3 Å, though we should mention that this barrier in the one-dimensional profile does not represent the true barrier of the physical process due to integration over the solvent degree of freedom (i.e., the coordination number CV in the FES shown in panel b). For the dissociated state above 4 Å, we can observe that there are some differences between runs. However, we can observe similar variance between the three reference runs from ref (57) as shown in Figure S9 in the SI. Therefore, it is difficult for us to say what the correct free energy profile is. Furthermore, our results in panel b are obtained at the end of the simulations and do not reflect that the bias—and thus the obtained FES—fluctuates during the simulation. Indeed, one of the main conclusions from Section 4.1 was that the fluctuations of the bias potential within individual runs were considerably smaller for the wavelets as compared to the polynomial basis functions.

**Figure 6:**
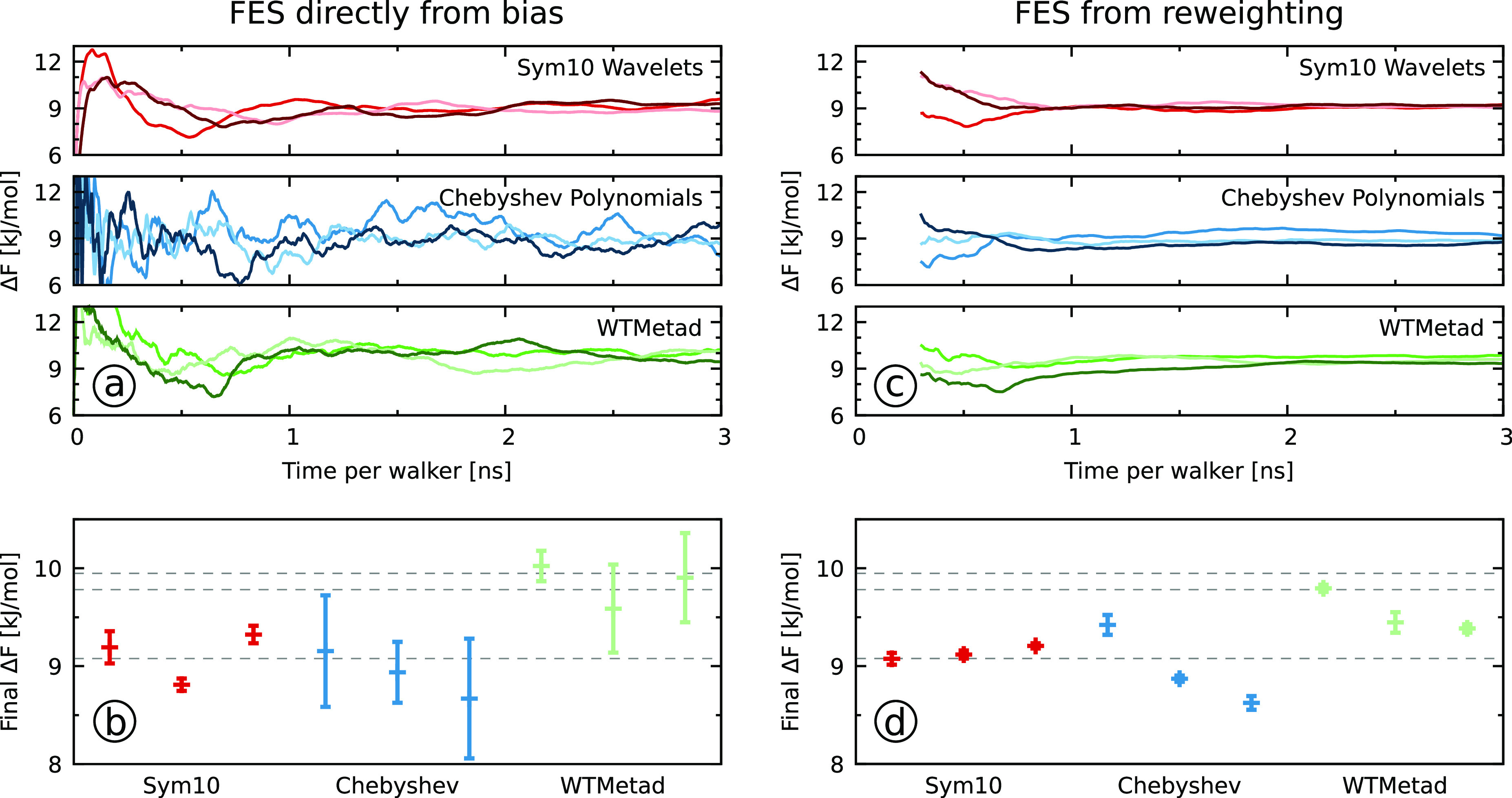
Results for the calcium carbonate system described in Section 3.4. (a, c) Time evolution of the free energy difference between the region with a Ca–C distance smaller 4 Å and the region with a Ca–C distance between 4 and 8 Å. For each biasing setup, we show three independent runs where the different color shades represent the individual runs. (b, d) The average of the free energy differences obtained over the last nanosecond by using 100 samples taken every 10 ps for each simulation. The error bars show the standard deviation to signify the quality of the individual measurements. We also show the results from ref (57) as black dotted lines. In panels a and b, we use the FES obtained directly from the bias via eq 9 (VES) or by summing over the deposited Gaussians (WTMetad). In panels c and d, we use the FES obtained through reweighting where we ignore the first 200 ps of each simulation.

To gauge the time evolution of the bias potential and FES, we consider the free energy difference between the contact ion-pair and loosely associated states of calcium carbonate. We select the region in CV space with a distance smaller than 4 Å as the contact ion-pair state and the region with distances between 4 and 8 Å as the loosely associated state and calculate the free energy difference according to eq 38. We note that this selection of the two regions does not necessarily coincide with the chemical definitions of ion association states.^57^ Here, we employ the free energy difference to monitor the stability of the bias potential and the obtained FES. In Figure 6a, we show the free energy difference obtained every 10 ps (simulation time per walker). For each of the biasing setups, we show the results from three independent runs.

In Figure 6a, we can see that the free energy differences obtained from the wavelet simulations converge faster and show less fluctuations than in the Chebyshev polynomial simulations. In particular, there are considerably larger fluctuations in the Chebyshev polynomial simulations. Furthermore, there is less difference between independent runs for the wavelets as compared to the Chebyshev polynomials. Therefore, when comparing the wavelets and the Chebyshev polynomials, we obtain the same conclusions as for the model system in Section 4.1: the wavelets exhibit less fluctuations of the bias potential within individual runs and less difference between different independent runs. The metadynamics simulations show a convergence behavior that is slightly worse than the wavelet simulations but still better than the Chebyshev polynomial simulations.

To further quantify the behavior of the simulations, we calculate the average and the standard deviation over the last nanosecond of each simulation and show the results in Figure 6b (numerical values are given in Table S1 in the SI). We chose the standard deviation because the time series from a single simulation is highly correlated and does not correspond to independent measurements. The standard deviation is thus shown as a measure of how much the free energy difference and thus also the bias fluctuate even at the end of the simulation. We can see that there is some spread in the averaged values, though all simulations agree with each other within 1 kJ/mol. We note that there is a similar spread in the three reference metadynamics simulations from ref (57) that are shown as black dotted lines in Figure 6b. Therefore, we cannot determine a reference value of the free energy difference. Noticeably, and consistent with the free energy differences time evolution in panel a, the wavelet simulations have the smallest standard deviation values, while the values are three to six times larger for the Chebyshev polynomial and metadynamics simulations.

From the results in panels a and b of Figure 6, we can conclude that the wavelets perform the best when considering the difference between independent simulations and fluctuations within runs.

So far, we have estimated the FES directly from the bias potential. An alternative way to obtain the FES is through reweighting. In fact, it is always a good practice to estimate the FES both directly from the bias potential and via reweighting and compare the results. The reweighting procedure assumes that the bias potential (i.e., the weights) is quasi-stationary. Therefore, we can expect the wavelets to perform better in this respect.

In panel c of Figure 6, we show the free energy difference values obtained from reweighted FESs every 10 ps. As before, we calculate the average and the standard deviation over the last nanosecond and present it in panel d of Figure 6, while numerical values are given in Table S1 in the SI. We can see that there are much smaller fluctuations in the free energy difference for all of the simulations as compared to panel b. All of the wavelet results agree well with each other and, when combined, yield a numerical estimate of 9.13 ± 0.04 kJ/mol (see Table S1 in the SI). There is more spread for the Chebyshev polynomial and the metadynamics simulations, but as before, all simulations agree within 1 kJ/mol. The reweighted metadynamics values tend to be lower than values obtained directly from the bias potential in panel b and closer to the wavelet results. As for the results obtained directly from the bias potential, we can conclude from the reweighted results that the wavelets perform the best when considering the difference between independent simulations and fluctuations within runs.

Overall, for the calcium carbonate association, we find that the wavelet basis functions exhibit an excellent performance. The wavelets result in a considerably better convergence behavior than the Chebyshev polynomials. The wavelet simulations also show a better convergence behavior than the metadynamics simulations.

## 5 Conclusions

In this work, we have introduced the usage of Daubechies wavelets as basis functions for variationally enhanced sampling. We have implemented the wavelets into the VES module of the PLUMED 2 code,^56^ tuned their parameters, and evaluated their performance on model systems and the calcium carbonate association process. Overall, the localized wavelet basis functions exhibit excellent performance and much more robust convergence behavior than the delocalized Chebyshev and Legendre polynomials used as basis functions within VES so far. In particular, the wavelet bases exhibit far smaller fluctuations of the bias potential within individual runs and smaller differences between independent runs. Less fluctuation of the bias potential is important when obtaining FESs and other equilibrium properties through reweighting as the reweighting procedure assumes a quasi-stationary bias potential. Based on our overall results, we can recommend wavelets as basis functions for variationally enhanced sampling.

We have also tested Gaussians and cubic B-splines as other types of localized basis functions. However, the Gaussian and the cubic B-spline basis functions perform worse than the wavelets for all the model systems in Section 4.1 and do not yield usable results for some systems. Therefore, we recommend against the usage of Gaussians and cubic B-splines as basis functions for VES.

One attractive feature of the wavelets basis functions is the multiresolution property displayed in eq 19. Starting with the father wavelets at some given scale, we can obtain a more accurate approximation of the FES by adding mother wavelets at finer scales. Here, we only employ a single level of father wavelets to expand the bias potential. An interesting future work would be to go beyond this and implement a multiresolution bias potential where we can increase the resolution on the fly during the simulation. Coupling this with a method to evaluate the quality of the current bias potential on the fly (for example, by using the effective sample size^38,85,91,92^) could allow us to automatically construct the VES bias potential with a predefined accuracy without the need to adapt the parameters manually.

Also, in the present work, we focused on a single type of wavelets, the family of Daubechies wavelets in their least asymmetric form. We also initially tested the Daubechies wavelets with an extremal phase. However, due to their noticeably worse performance than the symlets, we did not include them in this work’s extensive study. Nevertheless, other wavelet families could yield better performance for specific systems. For example, the boundary wavelets^93^ or the multiwavelets developed by Donovan et al.^94,95^ might be worthwhile to consider.

## Supporting Information Available

(S1) The effect of the type and scaling parameters for the Daubechies wavelet basis functions, (S2) the effect of the width parameter for the Gaussian basis functions, (S3) the Adam stochastic gradient descent algorithm, (S4) additional figures for the model potentials, (S5) the collective variables for the calcium carbonate system, (S6) additional figures for the calcium carbonate system, and (S7) numerical results for the calcium carbonate system (PDF)
Video illustrating the time evolution of the FES estimates of the VES method for different basis sets (MP4)

## References

[ref1] Dror R. O.; Dirks R. M.; Grossman J.; Xu H.; Shaw D. E. Biomolecular Simulation: A Computational Microscope for Molecular Biology. Annu. Rev. Biophys. 2012, 41, 429–452. 10.1146/annurev-biophys-042910-155245. 22577825

[ref2] Shaw D. E.; Adams P. J.; Azaria A.; Bank J. A.; Batson B.; Bell A.; Bergdorf M.; Bhatt J.; Butts J. A.; Correia T.; Dirks R. M.; Dror R. O.; Eastwood M. P.; Edwards B.; Even A.; Feldmann P.; Fenn M.; Fenton C. H.; Forte A.; Gagliardo J.; Gill G.; Gorlatova M.; Greskamp B.; Grossman J. P.; Gullingsrud J.; Harper A.; Hasenplaugh W.; Heily M.; Heshmat B. C.; Hunt J.; Ierardi D. J.; Iserovich L.; Jackson B. L.; Johnson N. P.; Kirk M. M.; Klepeis J. L.; Kuskin J. S.; Mackenzie K. M.; Mader R. J.; McGowen R.; McLaughlin A.; Moraes M. A.; Nasr M. H.; Nociolo L. J.; O’Donnell L.; Parker A.; Peticolas J. L.; Pocina G.; Predescu C.; Quan T.; Salmon J. K.; Schwink C.; Shim K. S.; Siddique N.; Spengler J.; Szalay T.; Tabladillo R.; Tartler R.; Taube A. G.; Theobald M.; Towles B.; Vick W.; Wang S. C.; Wazlowski M.; Weingarten M. J.; Williams J. M.; Yuh K. A. Anton 3: Twenty Microseconds of Molecular Dynamics Simulation before Lunch. In Proceedings of the International Conference for High Performance Computing, Networking, Storage and Analysis; Association for Computing Machinery, 2021.

[ref3] Phillips J. C.; Hardy D. J.; Maia J. D. C.; Stone J. E.; Ribeiro J. V.; Bernardi R. C.; Buch R.; Fiorin G.; Hénin J.; Jiang W.; McGreevy R.; Melo M. C. R.; Radak B. K.; Skeel R. D.; Singharoy A.; Wang Y.; Roux B.; Aksimentiev A.; Luthey-Schulten Z.; Kalé L. V.; Schulten K.; Chipot C.; Tajkhorshid E. Scalable Molecular Dynamics on CPU and GPU Architectures with NAMD. J. Chem. Phys. 2020, 153, 044130 10.1063/5.0014475. 32752662 PMC7395834

[ref4] Páll S.; Zhmurov A.; Bauer P.; Abraham M.; Lundborg M.; Gray A.; Hess B.; Lindahl E. Heterogeneous Parallelization and Acceleration of Molecular Dynamics Simulations in GROMACS. J. Chem. Phys. 2020, 153, 134110 10.1063/5.0018516. 33032406

[ref5] Khan H. N.; Hounshell D. A.; Fuchs E. R. H. Science and Research Policy at the End of Moore’s Law. Nat. Electron. 2018, 1, 14–21. 10.1038/s41928-017-0005-9.

[ref6] Dickson A.; Dinner A. R. Enhanced Sampling of Nonequilibrium Steady States. Annu. Rev. Phys. Chem. 2010, 61, 441–459. 10.1146/annurev.physchem.012809.103433. 20367083

[ref7] Chong L. T.; Saglam A. S.; Zuckerman D. M. Path-Sampling Strategies for Simulating Rare Events in Biomolecular Systems. Curr. Opin. Struct. Biol. 2017, 43, 88–94. 10.1016/j.sbi.2016.11.019. 27984811 PMC5420491

[ref8] Zuckerman D. M.; Chong L. T. Weighted Ensemble Simulation: Review of Methodology, Applications, and Software. Annu. Rev. Biophys. 2017, 46, 43–57. 10.1146/annurev-biophys-070816-033834. 28301772 PMC5896317

[ref9] Husic B. E.; Pande V. S. Markov State Models: From an Art to a Science. J. Am. Chem. Soc. 2018, 140, 2386–2396. 10.1021/jacs.7b12191. 29323881

[ref10] Allison J. R. Computational Methods for Exploring Protein Conformations. Biochem. Soc. Trans. 2020, 48, 1707–1724. 10.1042/BST20200193. 32756904 PMC7458412

[ref11] Kamenik A. S.; Linker S. M.; Riniker S. Enhanced Sampling without Borders: On Global Biasing Functions and How to Reweight Them. Phys. Chem. Chem. Phys. 2021, 24, 1225–1236. 10.1039/D1CP04809K. PMC8768491 34935813

[ref12] Hénin J.; Lelièvre T.; Shirts M. R.; Valsson O.; Delemotte L. Enhanced Sampling Methods for Molecular Dynamics Simulations. arXiv (Condensed Matter.Statistical Mechanics), February 8, 2022, 2202.04164, ver. 1. https://arxiv.org/abs/2202.04164 (accessed 2022-02-27).

[ref13] Fiorin G.; Klein M. L.; Hénin J. Using Collective Variables to Drive Molecular Dynamics Simulations. Mol. Phys. 2013, 111, 3345–3362. 10.1080/00268976.2013.813594.

[ref14] Giberti F.; Salvalaglio M.; Parrinello M. Metadynamics Studies of Crystal Nucleation. IUCrJ 2015, 2, 256–266. 10.1107/S2052252514027626. PMC4392418 25866662

[ref15] Pietrucci F. Strategies for the Exploration of Free Energy Landscapes: Unity in Diversity and Challenges Ahead. Rev. Phys. 2017, 2, 32–45. 10.1016/j.revip.2017.05.001.

[ref16] Wang Y.; Lamim Ribeiro J. M.; Tiwary P. Machine Learning Approaches for Analyzing and Enhancing Molecular Dynamics Simulations. Curr. Opin. Struct. Biol. 2020, 61, 139–145. 10.1016/j.sbi.2019.12.016. 31972477

[ref17] Noé F.; Tkatchenko A.; Müller K.-R.; Clementi C. Machine Learning for Molecular Simulation. Annu. Rev. Phys. Chem. 2020, 71, 361–390. 10.1146/annurev-physchem-042018-052331. 32092281

[ref18] Gkeka P.; Stoltz G.; Barati Farimani A.; Belkacemi Z.; Ceriotti M.; Chodera J. D.; Dinner A. R.; Ferguson A. L.; Maillet J.-B.; Minoux H.; Peter C.; Pietrucci F.; Silveira A.; Tkatchenko A.; Trstanova Z.; Wiewiora R.; Lelièvre T. Machine Learning Force Fields and Coarse-Grained Variables in Molecular Dynamics: Application to Materials and Biological Systems. J. Chem. Theory Comput. 2020, 16, 4757–4775. 10.1021/acs.jctc.0c00355. 32559068 PMC8312194

[ref19] Sidky H.; Chen W.; Ferguson A. L. Machine Learning for Collective Variable Discovery and Enhanced Sampling in Biomolecular Simulation. Mol. Phys. 2020, 118, e1737742 10.1080/00268976.2020.1737742.

[ref20] Torrie G. M.; Valleau J. P. Nonphysical Sampling Distributions in Monte Carlo Free-Energy Estimation: Umbrella Sampling. J. Comput. Phys. 1977, 23, 187–199. 10.1016/0021-9991(77)90121-8.

[ref21] Huber T.; Torda A. E.; van Gunsteren W. F. Local Elevation: A Method for Improving the Searching Properties of Molecular Dynamics Simulation. J. Comput.-Aided Mol. Des. 1994, 8, 695–708. 10.1007/BF00124016. 7738605

[ref22] Darve E.; Pohorille A. Calculating Free Energies Using Average Force. J. Chem. Phys. 2001, 115, 9169–9183. 10.1063/1.1410978.

[ref23] Comer J.; Gumbart J. C.; Hénin J.; Lelièvre T.; Pohorille A.; Chipot C. The Adaptive Biasing Force Method: Everything You Always Wanted to Know but Were Afraid to Ask. J. Phys. Chem. B 2015, 119, 1129–1151. 10.1021/jp506633n. 25247823 PMC4306294

[ref24] Lesage A.; Lelièvre T.; Stoltz G.; Hénin J. Smoothed Biasing Forces Yield Unbiased Free Energies with the Extended-System Adaptive Biasing Force Method. J. Phys. Chem. B 2017, 121, 3676–3685. 10.1021/acs.jpcb.6b10055. 27959559 PMC5402294

[ref25] Hansmann U. H. E.; Wille L. T. Global Optimization by Energy Landscape Paving. Phys. Rev. Lett. 2002, 88, 068105 10.1103/PhysRevLett.88.068105. 11863858

[ref26] Kästner J. Umbrella Sampling. Wiley Interdiscip. Rev.: Comput. Mol. Sci. 2011, 1, 932–942. 10.1002/wcms.66.

[ref27] Maragakis P.; van der Vaart A.; Karplus M. Gaussian-Mixture Umbrella Sampling. J. Phys. Chem. B 2009, 113, 4664–4673. 10.1021/jp808381s. 19284746 PMC2806548

[ref28] Warmflash A.; Bhimalapuram P.; Dinner A. R. Umbrella sampling for nonequilibrium processes. J. Chem. Phys. 2007, 127, 154112 10.1063/1.2784118. 17949137

[ref29] Laio A.; Parrinello M. Escaping Free-Energy Minima. Proc. Natl. Acad. Sci. U. S. A. 2002, 99, 12562–12566. 10.1073/pnas.202427399. 12271136 PMC130499

[ref30] Barducci A.; Bussi G.; Parrinello M. Well-Tempered Metadynamics: A Smoothly Converging and Tunable Free-Energy Method. Phys. Rev. Lett. 2008, 100, 020603 10.1103/PhysRevLett.100.020603. 18232845

[ref31] Valsson O.; Tiwary P.; Parrinello M. Enhancing Important Fluctuations: Rare Events and Metadynamics from a Conceptual Viewpoint. Annu. Rev. Phys. Chem. 2016, 67, 159–184. 10.1146/annurev-physchem-040215-112229. 26980304

[ref32] Dama J. F.; Hocky G. M.; Sun R.; Voth G. A. Exploring Valleys without Climbing Every Peak: More Efficient and Forgiving Metabasin Metadynamics via Robust On-the-Fly Bias Domain Restriction. J. Chem. Theory Comput. 2015, 11, 5638–5650. 10.1021/acs.jctc.5b00907. 26587809 PMC4675329

[ref33] Pfaendtner J.; Bonomi M. Efficient Sampling of High-Dimensional Free-Energy Landscapes with Parallel Bias Metadynamics. J. Chem. Theory Comput. 2015, 11, 5062–5067. 10.1021/acs.jctc.5b00846. 26574304

[ref34] Whitmer J. K.; Chiu C.-c.; Joshi A. A.; de Pablo J. J. Basis Function Sampling: A New Paradigm for Material Property Computation. Phys. Rev. Lett. 2014, 113, 190602 10.1103/PhysRevLett.113.190602. 25415892

[ref35] Whitmer J. K.; Fluitt A. M.; Antony L.; Qin J.; McGovern M.; de Pablo J. J. Sculpting Bespoke Mountains: Determining Free Energies with Basis Expansions. J. Chem. Phys. 2015, 143, 044101 10.1063/1.4927147. 26233101

[ref36] Sidky H.; Whitmer J. K. Learning Free Energy Landscapes Using Artificial Neural Networks. J. Chem. Phys. 2018, 148, 104111 10.1063/1.5018708. 29544298

[ref37] Ribeiro J. M. L.; Bravo P.; Wang Y.; Tiwary P. Reweighted autoencoded variational Bayes for enhanced sampling (RAVE). J. Chem. Phys. 2018, 149, 072301 10.1063/1.5025487. 30134694

[ref38] Invernizzi M.; Parrinello M. Rethinking Metadynamics: From Bias Potentials to Probability Distributions. J. Phys. Chem. Lett. 2020, 11, 2731–2736. 10.1021/acs.jpclett.0c00497. 32191470

[ref39] Invernizzi M.; Piaggi P. M.; Parrinello M. Unified Approach to Enhanced Sampling. Phys. Rev. X 2020, 10, 041034 10.1103/PhysRevX.10.041034.

[ref40] Giberti F.; Tribello G. A.; Ceriotti M. Global Free-Energy Landscapes as a Smoothly Joined Collection of Local Maps. J. Chem. Theory Comput. 2021, 17, 3292–3308. 10.1021/acs.jctc.0c01177. 34003008

[ref41] Bal K. M. Reweighted Jarzynski Sampling: Acceleration of Rare Events and Free Energy Calculation with a Bias Potential Learned from Nonequilibrium Work. J. Chem. Theory Comput. 2021, 17, 6766–6774. 10.1021/acs.jctc.1c00574. 34714088

[ref42] Valsson O.; Parrinello M. Variational Approach to Enhanced Sampling and Free Energy Calculations. Phys. Rev. Lett. 2014, 113, 090601 10.1103/PhysRevLett.113.090601. 25215968

[ref43] Valsson O.; Parrinello M. Variationally Enhanced Sampling. In Handbook of Materials Modeling: Methods: Theory and Modeling; Andreoni W., Yip S., Eds.; Springer: Cham, Switzerland, 2020, 621–634.

[ref44] Bonati L.; Zhang Y.-Y.; Parrinello M. Neural Networks-Based Variationally Enhanced Sampling. Proc. Natl. Acad. Sci. U. S. A. 2019, 116, 17641–17647. 10.1073/pnas.1907975116. 31416918 PMC6731643

[ref45] Piaggi P. M.; Valsson O.; Parrinello M. A Variational Approach to Nucleation Simulation. Faraday Discuss. 2016, 195, 557–568. 10.1039/C6FD00127K. 27752683

[ref46] McCarty J.; Valsson O.; Parrinello M. Bespoke Bias for Obtaining Free Energy Differences within Variationally Enhanced Sampling. J. Chem. Theory Comput. 2016, 12, 2162–2169. 10.1021/acs.jctc.6b00125. 27057791

[ref47] Invernizzi M.; Valsson O.; Parrinello M. Coarse Graining from Variationally Enhanced Sampling Applied to the Ginzburg–Landau Model. Proc. Natl. Acad. Sci. U. S. A. 2017, 114, 3370–3374. 10.1073/pnas.1618455114. 28292890 PMC5380034

[ref48] Invernizzi M.; Parrinello M. Making the Best of a Bad Situation: A Multiscale Approach to Free Energy Calculation. J. Chem. Theory Comput. 2019, 15, 2187–2194. 10.1021/acs.jctc.9b00032. 30822383

[ref49] McCarty J.; Valsson O.; Tiwary P.; Parrinello M. Variationally Optimized Free-Energy Flooding for Rate Calculation. Phys. Rev. Lett. 2015, 115, 070601 10.1103/PhysRevLett.115.070601. 26317704

[ref50] Demuynck R.; Rogge S. M. J.; Vanduyfhuys L.; Wieme J.; Waroquier M.; Van Speybroeck V. Efficient Construction of Free Energy Profiles of Breathing Metal—Organic Frameworks Using Advanced Molecular Dynamics Simulations. J. Chem. Theory Comput. 2017, 13, 5861–5873. 10.1021/acs.jctc.7b01014. 29131647 PMC5729547

[ref51] Demuynck R.; Wieme J.; Rogge S. M. J.; Dedecker K. D.; Vanduyfhuys L.; Waroquier M.; Van Speybroeck V. Protocol for Identifying Accurate Collective Variables in Enhanced Molecular Dynamics Simulations for the Description of Structural Transformations in Flexible Metal–Organic Frameworks. J. Chem. Theory Comput. 2018, 14, 5511–5526. 10.1021/acs.jctc.8b00725. 30336016 PMC6236469

[ref52] Daubechies I. Orthonormal Bases of Compactly Supported Wavelets. Commun. Pure Appl. Math. 1988, 41, 909–996. 10.1002/cpa.3160410705.

[ref53] Mohr S.; Ratcliff L. E.; Boulanger P.; Genovese L.; Caliste D.; Deutsch T.; Goedecker S. Daubechies Wavelets for Linear Scaling Density Functional Theory. J. Chem. Phys. 2014, 140, 204110 10.1063/1.4871876. 24880269

[ref54] Ratcliff L. E.; Dawson W.; Fisicaro G.; Caliste D.; Mohr S.; Degomme A.; Videau B.; Cristiglio V.; Stella M.; D’Alessandro M.; Goedecker S.; Nakajima T.; Deutsch T.; Genovese L. Flexibilities of Wavelets as a Computational Basis Set for Large-Scale Electronic Structure Calculations. J. Chem. Phys. 2020, 152, 194110 10.1063/5.0004792. 33687268

[ref55] Maiolo M.; Vancheri A.; Krause R.; Danani A. Wavelets as Basis Functions to Represent the Coarse-Graining Potential in Multiscale Coarse Graining Approach. J. Comput. Phys. 2015, 300, 592–604. 10.1016/j.jcp.2015.07.039.

[ref56] Tribello G. A.; Bonomi M.; Branduardi D.; Camilloni C.; Bussi G. PLUMED 2: New Feathers for an Old Bird. Comput. Phys. Commun. 2014, 185, 604–613. 10.1016/j.cpc.2013.09.018.

[ref57] Kellermeier M.; Raiteri P.; Berg J. K.; Kempter A.; Gale J. D.; Gebauer D. Entropy Drives Calcium Carbonate Ion Association. ChemPhysChem 2016, 17, 3535–3541. 10.1002/cphc.201600653. 27540706

[ref58] Valsson O.; Parrinello M. Well-Tempered Variational Approach to Enhanced Sampling. J. Chem. Theory Comput. 2015, 11, 1996–2002. 10.1021/acs.jctc.5b00076. 26574405

[ref59] Tiwary P.; Parrinello M. A Time-Independent Free Energy Estimator for Metadynamics. J. Phys. Chem. B 2015, 119, 736–742. 10.1021/jp504920s. 25046020

[ref60] Bach F.; Moulines E. Non-Strongly-Convex Smooth Stochastic Approximation with Convergence Rate *O*(1/*n*). In Advances in Neural Information Processing Systems 26; Curran Associates, 2013; pp 773–781.

[ref61] Boyd J. P. Chebyshev and Fourier Spectral Methods, 2nd ed.; Dover Publications: Mineola, NY, 2001.

[ref62] Daubechies I. Ten Lectures on Wavelets; CBMS-NSF Regional Conference Series in Applied Mathematics 61; Society for Industrial and Applied Mathematics: Philadelphia, PA, 1992.

[ref63] Goedecker S. Wavelets and Their Application for the Solution of Partial Differential Equations in Physics; Presses Polytechniques et Universitaires Romandes: Lausanne, Switzerland, 1998.

[ref64] Baftizadeh F.; Cossio P.; Pietrucci F.; Laio A. Protein Folding and Ligand-Enzyme Binding from Bias-Exchange Metadynamics Simulations. Curr. Phys. Chem. 2012, 2, 79–91. 10.2174/1877946811202010079.

[ref65] Crespo Y.; Marinelli F.; Pietrucci F.; Laio A. Metadynamics Convergence Law in a Multidimensional System. Phys. Rev. E 2010, 81, 055701(R) 10.1103/PhysRevE.81.055701. 20866290

[ref66] McGovern M.; de Pablo J. A Boundary Correction Algorithm for Metadynamics in Multiple Dimensions. J. Chem. Phys. 2013, 139, 084102 10.1063/1.4818153. 24006969

[ref67] Habermann C.; Kindermann F. Multidimensional Spline Interpolation: Theory and Applications. Comput. Econ. 2007, 30, 153–169. 10.1007/s10614-007-9092-4.

[ref68] Promoting Transparency and Reproducibility in Enhanced Molecular Simulations. Nat. Methods 2019, 16, 670–673. 10.1038/s41592-019-0506-8. 31363226

[ref69] Strang G.; Nguyen T. Wavelets and Filter Banks, 2nd ed.; Wellesley-Cambridge Press: Wellesley, MA, 1997.

[ref70] Bussi G.; Parrinello M. Accurate Sampling Using Langevin Dynamics. Phys. Rev. E 2007, 75, 056707 10.1103/PhysRevE.75.056707. 17677198

[ref71] Wolfe S.; Schlegel H. B.; Csizmadia I. G.; Bernardi F. Chemical Dynamics of Symmetric and Asymmetric Reaction Coordinates. J. Am. Chem. Soc. 1975, 97, 2020–2024. 10.1021/ja00841a005.

[ref72] Quapp W. A Growing String Method for the Reaction Pathway Defined by a Newton Trajectory. J. Chem. Phys. 2005, 122, 174106 10.1063/1.1885467. 15910022

[ref73] Kingma D. P.; Ba J. Adam: A Method for Stochastic Optimization. In 3rd International Conference on Learning Representations; ISCA, 2015.

[ref74] Plimpton S. Fast Parallel Algorithms for Short-Range Molecular Dynamics. J. Comput. Phys. 1995, 117, 1–19. 10.1006/jcph.1995.1039.

[ref75] Demichelis R.; Raiteri P.; Gale J. D.; Quigley D.; Gebauer D. Stable Prenucleation Mineral Clusters Are Liquid-like Ionic Polymers. Nat. Commun. 2011, 2, 590 10.1038/ncomms1604. 22186886 PMC3247826

[ref76] Raiteri P.; Demichelis R.; Gale J. D. Thermodynamically Consistent Force Field for Molecular Dynamics Simulations of Alkaline-Earth Carbonates and Their Aqueous Speciation. J. Phys. Chem. C 2015, 119, 24447–24458. 10.1021/acs.jpcc.5b07532.

[ref77] Wu Y.; Tepper H. L.; Voth G. A. Flexible Simple Point-Charge Water Model with Improved Liquid-State Properties. J. Chem. Phys. 2006, 124, 024503 10.1063/1.2136877. 16422607

[ref78] Nosé S. A Unified Formulation of the Constant Temperature Molecular Dynamics Methods. J. Chem. Phys. 1984, 81, 511–519. 10.1063/1.447334.

[ref79] Hoover W. G. Canonical Dynamics: Equilibrium Phase-Space Distributions. Phys. Rev. A 1985, 31, 1695–1697. 10.1103/PhysRevA.31.1695. 9895674

[ref80] Tuckerman M. E.; Alejandre J.; López-Rendón R.; Jochim A. L.; Martyna G. J. A Liouville-operator Derived Measure-Preserving Integrator for Molecular Dynamics Simulations in the Isothermal–Isobaric Ensemble. J. Phys. A: Math. Gen. 2006, 39, 5629–5651. 10.1088/0305-4470/39/19/S18.

[ref81] Hockney R. W.; Eastwood J. W. Computer Simulation Using Particles; CRC Press: Boca Raton, FL, 1988.

[ref82] Raiteri P.; Laio A.; Gervasio F. L.; Micheletti C.; Parrinello M. Efficient Reconstruction of Complex Free Energy Landscapes by Multiple Walkers Metadynamics. J. Phys. Chem. B 2006, 110, 3533–3539. 10.1021/jp054359r. 16494409

[ref83] Branduardi D.; Bussi G.; Parrinello M. Metadynamics with Adaptive Gaussians. J. Chem. Theory Comput. 2012, 8, 2247–2254. 10.1021/ct3002464. 26588957

[ref84] Coveney P. V.; Wan S. On the Calculation of Equilibrium Thermodynamic Properties from Molecular Dynamics. Phys. Chem. Chem. Phys. 2016, 18, 30236–30240. 10.1039/C6CP02349E. 27165501

[ref85] Grossfield A.; Patrone P. N.; Roe D. R.; Schultz A. J.; Siderius D.; Zuckerman D. M. Best Practices for Quantification of Uncertainty and Sampling Quality in Molecular Simulations. LiveCoMS 2019, 1, 5067 10.33011/livecoms.1.1.5067. PMC6286151 30533602

[ref86] Pampel B.; Valsson O. Improving the Efficiency of Variationally Enhanced Sampling with Wavelet-Based Bias Potentials (v1.0) [Data set]. Zenodo, 2022. 10.5281/zenodo.5851773. PMC9281396 35762642

[ref87] Aguilar-Mogas A.; Giménez X.; Bofill J. M. Implementation of an Algorithm Based on the Runge-Kutta-Fehlberg Technique and the Potential Energy as a Reaction Coordinate to Locate Intrinsic Reaction Paths. J. Comput. Chem. 2010, 2510–2525. 10.1002/jcc.21539. 20652993

[ref88] Bofill J. M.; Quapp W.; Caballero M. Locating Transition States on Potential Energy Surfaces by the Gentlest Ascent Dynamics. Chem. Phys. Lett. 2013, 583, 203–208. 10.1016/j.cplett.2013.07.074.

[ref89] Zhang X.-J.; Shang C.; Liu Z.-P. Double-Ended Surface Walking Method for Pathway Building and Transition State Location of Complex Reactions. J. Chem. Theory Comput. 2013, 9, 5745–5753. 10.1021/ct4008475. 26592302

[ref90] Debnath J.; Parrinello M. Gaussian Mixture-Based Enhanced Sampling for Statics and Dynamics. J. Phys. Chem. Lett. 2020, 11, 5076–5080. 10.1021/acs.jpclett.0c01125. 32510225

[ref91] Zhang X.; Bhatt D.; Zuckerman D. M. Automated Sampling Assessment for Molecular Simulations Using the Effective Sample Size. J. Chem. Theory Comput. 2010, 6, 3048–3057. 10.1021/ct1002384. 21221418 PMC3017371

[ref92] Martino L.; Elvira V.; Louzada F. Effective Sample Size for Importance Sampling Based on Discrepancy Measures. Signal Process. 2017, 131, 386–401. 10.1016/j.sigpro.2016.08.025.

[ref93] Bertoluzza S.; Falletta S. Building Wavelets on 0,1 at Large Scales. J. Fourier Anal. Appl. 2003, 9, 261–288. 10.1007/s00041-003-0014-0.

[ref94] Donovan G. C.; Geronimo J. S.; Hardin D. P. Orthogonal Polynomials and the Construction of Piecewise Polynomial Smooth Wavelets. SIAM J. Math. Anal. 1999, 30, 1029–1056. 10.1137/S0036141096313112.

[ref95] Donovan G. C.; Geronimo J. S.; Hardin D. P. Intertwining Multiresolution Analyses and the Construction of Piecewise-Polynomial Wavelets. SIAM J. Math. Anal. 1996, 27, 1791–1815. 10.1137/S0036141094276160.

