# Supplementary material for: Improving the Efficiency of Variationally Enhanced Sampling with Wavelet-Based Bias Potentials
Source: J Chem Theory Comput. 2022 Jun 28;18(7):4127–41. doi: 10.1021/acs.jctc.2c00197 (PMC9281396; doi:10.1021/acs.jctc.2c00197)
Supplement: Supplementary file 1 — ct2c00197_si_001.pdf [file ct2c00197_si_001.pdf]

Supporting Information: Improving the Efficiency of Variationally Enhanced Sampling with Wavelet-Based Bias Potentials

Benjamin Pampel† and Omar Valsson*,†,‡

†*Max Planck Institute for Polymer Research, Ackermannweg 10, D-55128 Mainz, Germany*

‡*Present Address: Department of Chemistry, University of North Texas, 1508 W Mulberry St, Denton, TX 76201, USA. E-mail: omar.valsson@unt.edu*

E-mail: valsson@mpip-mainz.mpg.de

S1 Daubechies Wavelets Basis Functions: Effect of Type and Scaling Parameters

As discussed in Section 2.4 in the main text, two parameters can be adjusted when using a wavelet basis in VES. In this work, we use only the single level of father wavelets to form the basis set, so when the term wavelet is used, we always refer to these.

The first parameter of the basis is the type of wavelet functions to be used. For wavelets forming orthogonal bases the two most common families are the ones with the highest regularity called "Daubechies Wavelets" (or "maximum phase"), and the ones with the least asymmetry called "Symlets". We initially tested both types and found the difference in regularity less important for good convergence than the symmetry of the functions and decided

to consider only wavelets of the Symlet family. The members of the family are characterized by the number of vanishing moments N. Father Wavelets with N vanishing moments can locally represent polynomials up to order N well, so steeper slopes require wavelets with more vanishing moments. With increasing vanishing moments also the regularity of the wavelet increases, at the cost of a larger range at which they are non-zero if used at the same scale. These properties can be seen in Figure S1, where bases constructed by different Symlets can be seen.

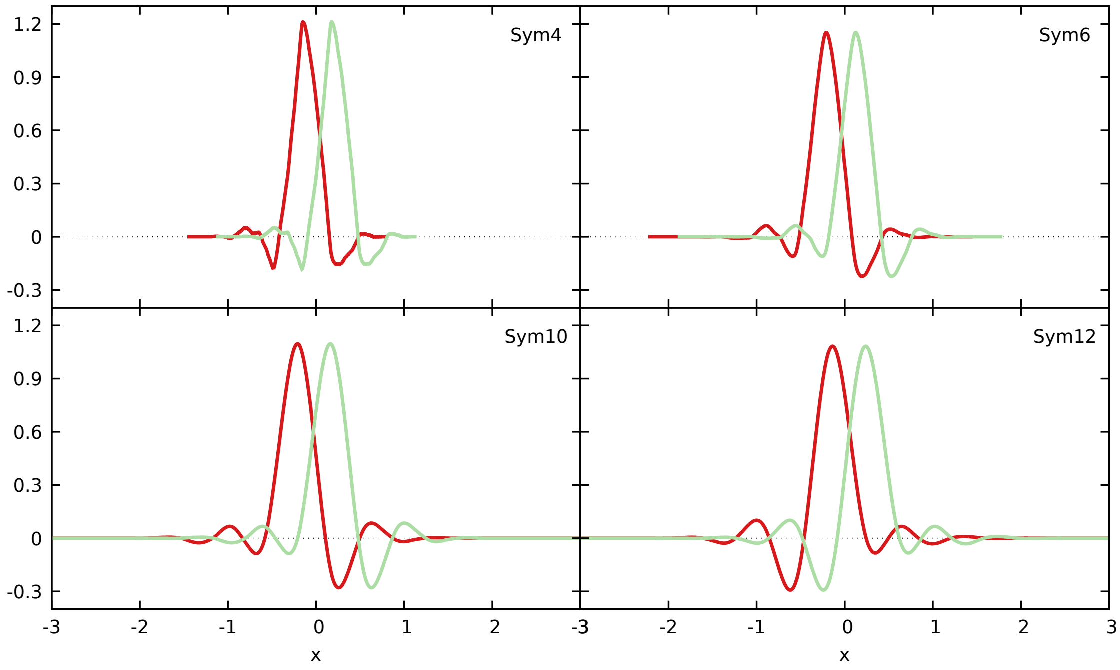

Figure S1: Visualization of different types of Symlet bases. The Sym8 wavelets can be seen in Figure 1 in the main text. Each basis consists of shifted functions of the same type. Per basis set only two adjacent functions are shown, while the complete basis includes all shifted wavelets in the given range. Visible is the increasing length on which the individual wavelet functions are defined, from only slightly over 2 units to more than the full CV range of 6 units. This results in more overlapping functions when using wavelet with more vanishing moments, which makes more "irregular" features, i.e. features of higher polynomial order, representable. Also, the regularity of the wavelet increases from rougher spikes at Sym4 to smooth wave-like "oscillations" at Sym12.

We tested the performance of different Symlet types on the one-dimensional double-well potential from Section 4.1 of the main text, using the same simulation protocol as described in Section 3.1. The results can be seen in Figure S2. Noticeable is that there is a minimum

number of vanishing moments N needed to properly represent the underlying free energy surface of the system. While Sym4 wavelets result in only a very coarse approximation, starting at Sym6 there are only minimal differences to be seen. This minimum number of vanishing moments depends on the shape of the free energy and can therefore vary. Based on these results we chose Sym8 wavelets for the benchmark simulations in the paper.

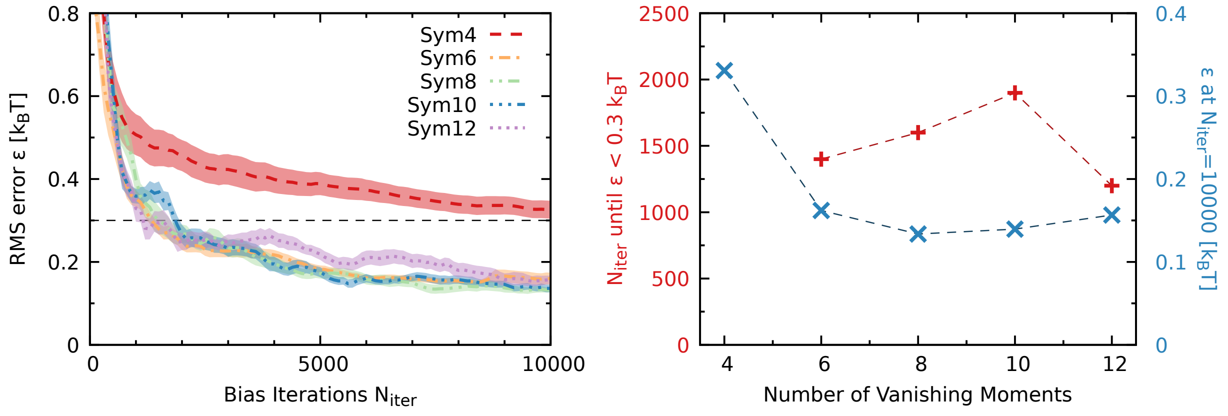

Figure S2: *Left panel*: Root mean square error of the free energy calculated directly from the bias potential for the one-dimensional double-well system (see Sections 3.1 and 4.1 in main text) for different wavelet types as a function of time. For each line, 20 independent simulations with 22 basis functions of a specific wavelet type were run, the form of the different functions can be seen in Figure S1. The shaded areas show the standard error. The black dashed line denotes the error threshold of $\epsilon = 0.3\,k_{\mathrm{B}}T$.
Right panel: Required number of bias iterations until an RMS error of less than $0.3\,k_{\mathrm{B}}T$ is reached, together with the final error after 10.000 iterations. The number of iterations is missing for Sym4 wavelets, because the required accuracy was not reached. With increasing number of vanishing moments, the individual basis functions overlap more and are thus able to represent features of higher polynomial order. For this simple system, there are no visible differences in the performance Sym6, while Sym4 wavelets are not able to represent the free energy as accurately. The standard deviation of the error does not show substantial differences between the different basis functions.

Secondly, the scaling j of the individual basis functions can be chosen. Instead of selecting the scale directly, we set the desired number of basis functions in the range of the bias potential. Using more basis functions allows representing finer features better. We ran simulations with different numbers of Sym8 basis functions for the one-dimensional double-well potential from Section 4.1 of the main text, using the same simulation protocol as described in Section 3.1. The resulting time evolution of the root mean square error is

shown in Figure S3. Similar to before, we notice that there is a minimum number of basis

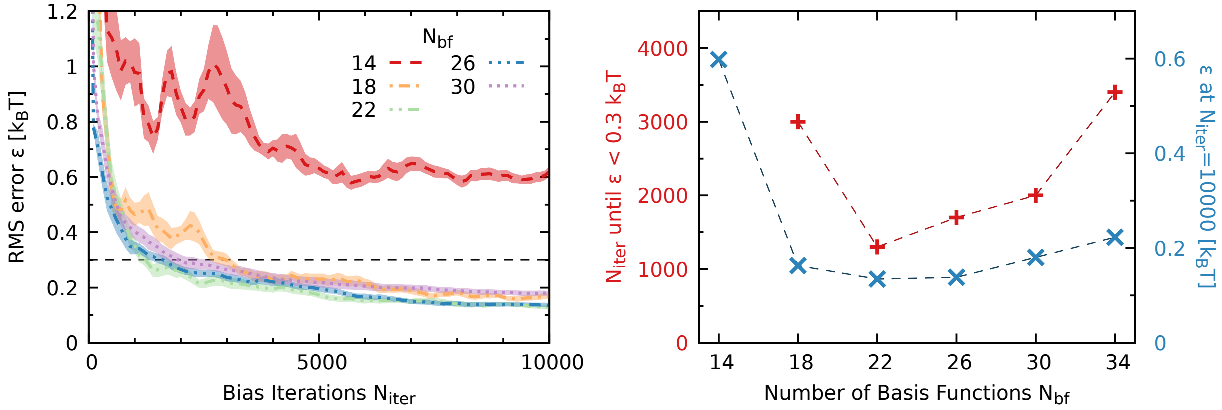

Figure S3: *Left panel*: Root mean square error of the free energy calculated directly from the bias potential for the one-dimensional double-well system (see Sections 3.1 and 4.1 in main text) for different number of Sym8 wavelet basis functions as a function of time. For each line, 20 analog simulations with the specified number of basis functions were conducted, the shaded areas show the standard error. The number of basis functions is adjusted by scaling the individual wavelets in CV space. The black dashed line denotes the error threshold of $\epsilon = 0.3\, k_{\mathrm{B}}T$.
Right panel: Required number of bias iterations until an RMS error of less than 0.3 $k_{\mathrm{B}}T$ is reached, together with the final error after 10.000 iterations. The number of iterations is missing for 14 basis functions, because the required accuracy was not reached. This shows that there is a minimum number of basis functions required to adequately represent the underlying free energy surface. A good representation is possible starting at 18 basis functions but even better with 22. Adding more basis functions does not improve the situation further. On the contrary, the introduction of significantly more optimization parameters complicates the optimization and therefore slows convergence. This can be seen from the increasing required number of iterations for larger number of basis functions.

functions required to represent the system well, in this case, it is around 20. Additionally, we note that using more basis functions leads to a higher number of coefficients to be optimized. Adding more basis functions after the basis can already adequately represent the features of the system might thus lead to worse performance. This is visible in the higher number of required iterations if 30 or more basis functions are used, although the final RMS error does not significantly differ. In general, using too many basis functions is better than using too few.

S2 Gaussian Basis Functions: Effect of Width Parameter

As discussed in Section 2.5 in the main text, in principle, the width parameter σ of Gaussian functions (as defined in eq. 23) can be adjusted freely. To evaluate the influence of different choices we performed simulations on the one-dimensional double-well potential from Section 4.1 of the main text, using the same simulation protocol as described in Section 3.1. We only consider basis sets with the same width parameter for all Gaussian functions. Results for different widths of the Gaussians can be seen in Figure S4. We find that the choice of ref.

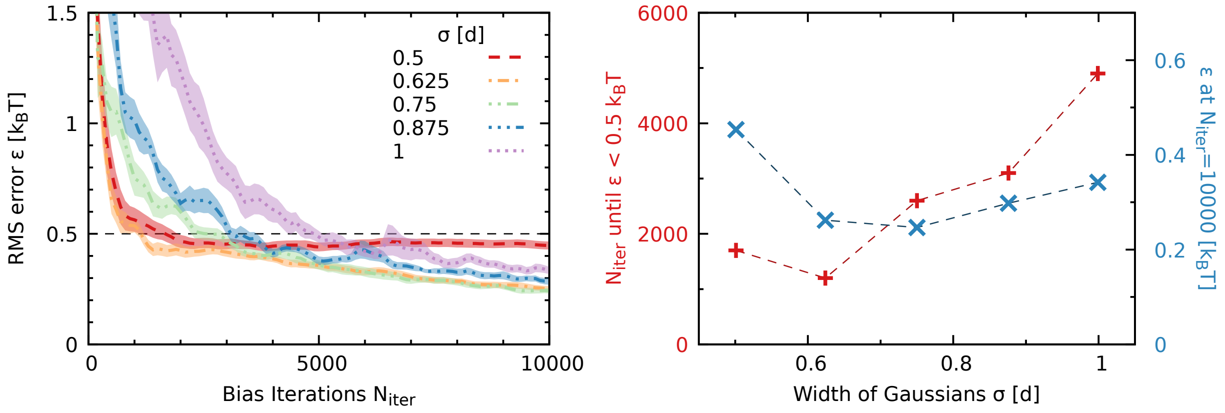

Figure S4: *Left panel*: Root mean square error of the free energy calculated directly from the bias potential for the one-dimensional double-well system (see Sections 3.1 and 4.1 in main text) for different values of the width parameter σ as a function of time. The lines denote the average over 20 runs and the shaded area the corresponding standard error. The values of the width parameter are given as fractions of the distance between the centers of the Gaussians d. The black dashed line denotes the error threshold of $\epsilon = 0.5\,k_\mathrm{B}T$.
Right panel: Required number of bias iterations until an RMS error of less than 0.3 $k_\mathrm{B}T$ is reached, together with the final error after 10.000 iterations. $\sigma = d$ is the choice of Ref. S1 and $\sigma = 0.5d$ results in a only slightly larger overlap than the FWHM. It can be seen that a too small choice ($\sigma = 0.5d$) results in a basis set that is only able to yield a very rough representation of the underlying FES. On the other hand, the basis set consisting of the widest Gaussians needs more iterations to reach the same quality of approximation as for the intermediate choices.

S1, choosing the width parameter equal to the distance between basis functions, is working well but can be improved. Of note is that for this choice in 2 out of the 20 simulations

the bias did not converge correctly. As non-converged simulations would largely impact the values of error measures, these were excluded and replaced by new simulations. We could not observe similar problems with narrower Gaussians. On the contrary, choosing a smaller width parameter results in a better approximation at short times and less fluctuation between individual runs. After sufficiently many iterations, here in the order of 10,000, the differences are becoming negligible. On the other hand, a too small parameter results in a basis not capable of representing the underlying features at all. This can be seen when using a width parameter as small as half the distance between Gaussians. For this choice the final approximation of the basis set is significantly less accurate. Even narrower Gaussians result in systematically worse performance and would be quickly out of the given range in Figure S4a), so they were omitted. We conclude that a value of σ between 5/8 and 7/8 of the distance between the functions gives best results for this system. Within that range, the results are robust with respect to parameter changes. For the comparison with other basis functions, we choose a value of σ equal to 3/4 of the distance between the functions.

S3 Adam Stochastic Gradient Descent Algorithm

The "adaptive moment estimation" (Adam) algorithm[S2] is one of the most popular optimization algorithms for machine learning applications. We use it as an alternative to Bach's averaged stochastic gradient descent to update the coefficients of the bias potential. It uses a running average $\boldsymbol{m}$ and second moment $\boldsymbol{v}$ of the gradient $\boldsymbol{g} = \nabla\Omega(\boldsymbol{\alpha})$, where at iteration n these are updated with the current value of the gradient according to

$$\begin{aligned} \boldsymbol{m}^{(n)} &= \beta_1 \boldsymbol{m}^{(n-1)} + (1-\beta_1)\boldsymbol{g}^{(n)} \\ \boldsymbol{v}^{(n)} &= \beta_2 \boldsymbol{v}^{(n-1)} + (1-\beta_2)\boldsymbol{g}^{(n)}\boldsymbol{g}^{(n)} \end{aligned}$$

The parameters β_1 and β_2 determine the "memory" of the averaging process, we set them to the literature values of $\beta_1 = 0.9$ and $\beta_2 = 0.999$. The update rule for the coefficients is

then given by

$$\boldsymbol{\alpha}^{(n)} = \boldsymbol{\alpha}^{(n-1)} - \frac{\eta}{\sqrt{\boldsymbol{v}^{(n)}} + \epsilon}\boldsymbol{m}^{(n)} \tag{S1}$$

where $\epsilon = 10^{-8}$ is a small parameter preventing division by zero and η is a stepsize to be set by the user. The algorithm scales the stepsize by the variance, thus coefficients with larger fluctuations get updated in smaller steps. We found the literature value of $\eta = 0.001$ to small for an efficient convergence and instead chose $\eta = 0.005$.

As $\boldsymbol{m}$ and $\boldsymbol{v}$ are initialized to zero, the algorithm would be biased towards that value in the first few steps. To overcome this, we used the correction proposed with the algorithm, for further details see Ref. S2.

S4 Model Potentials: Additional Figures

While in the paper only the averages and standard error of the error measure are shown, in Figure S5 we exemplary show the RMS error for individual runs for the one-dimensional double-well system described in Section 3.1 and analyzed in Section 4.1 of the main text. Visible are higher fluctuations for the simulations with Legendre polynomials both within and between the runs, which we find to be in accordance with the conclusions drawn in the main text.

Figure S6 shows the same plot as Figure 2**b** of the main text, but plotted on logarithmic axes for both the RMS Error and the number of iterations. Clearly visible is the near-linear decrease of the RMS error in this representation, which denotes an convergence behavior according to a power law.

In Figure S7**a** the error measure of the FES is given for the two-dimensional Wolfe-Quapp potential. Simulations with cubic splines fail to converge to a good approximation of the correct free energy here. Of the other used basis functions, wavelets give the best result. They give a good approximation already after a few thousand iterations and give also the best approximation after the full 10,000 iterations. Gaussians and Legendre polynomials result

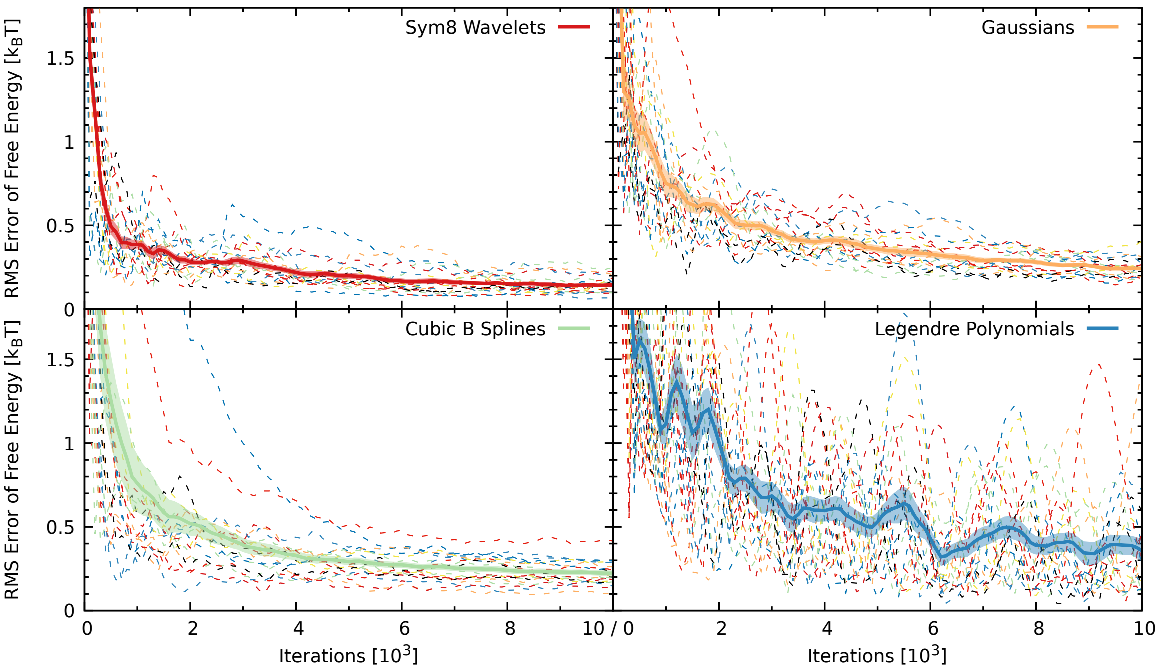

Figure S5: The RMS Error of the FES calculated directly from the bias potential for the one-dimensional double-well system (see Sections 3.1 and 4.1 in main text) as a function of the number of bias iterations for different basis functions. Each dashed line gives the result for an individual run, while the solid lines denote the average over the 20 runs and the shaded area the corresponding standard error.

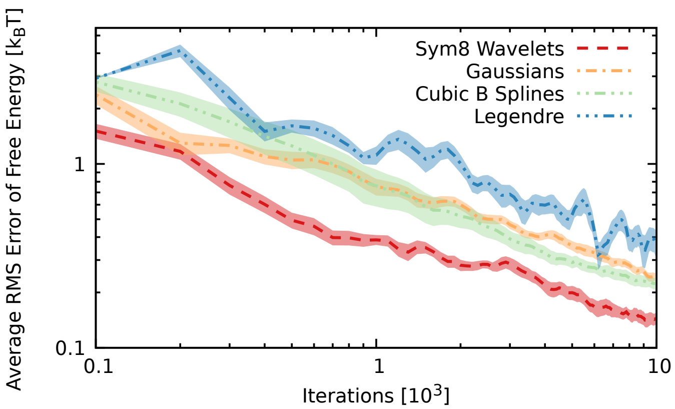

Figure S6: The average and standard deviation of the RMS Error of the FES calculated directly from the bias potential for the one-dimensional double-well system (see Sections 3.1 and 4.1 in main text) as a function of the number of bias iterations for different basis functions plotted over logarithmic axes. See Figure 2**b** of the main text for the same data plotted on linear axes.

in an only slightly higher error at the end of the simulation but take considerably more iterations to reach a good approximation. Although both curves look similar, Gaussian

basis functions are better at short times compared to the Legendre polynomials. Also the fluctuations between runs are significantly smaller for wavelets and Gaussians than for the Legendre polynomials, which can be seen in the smaller standard error.

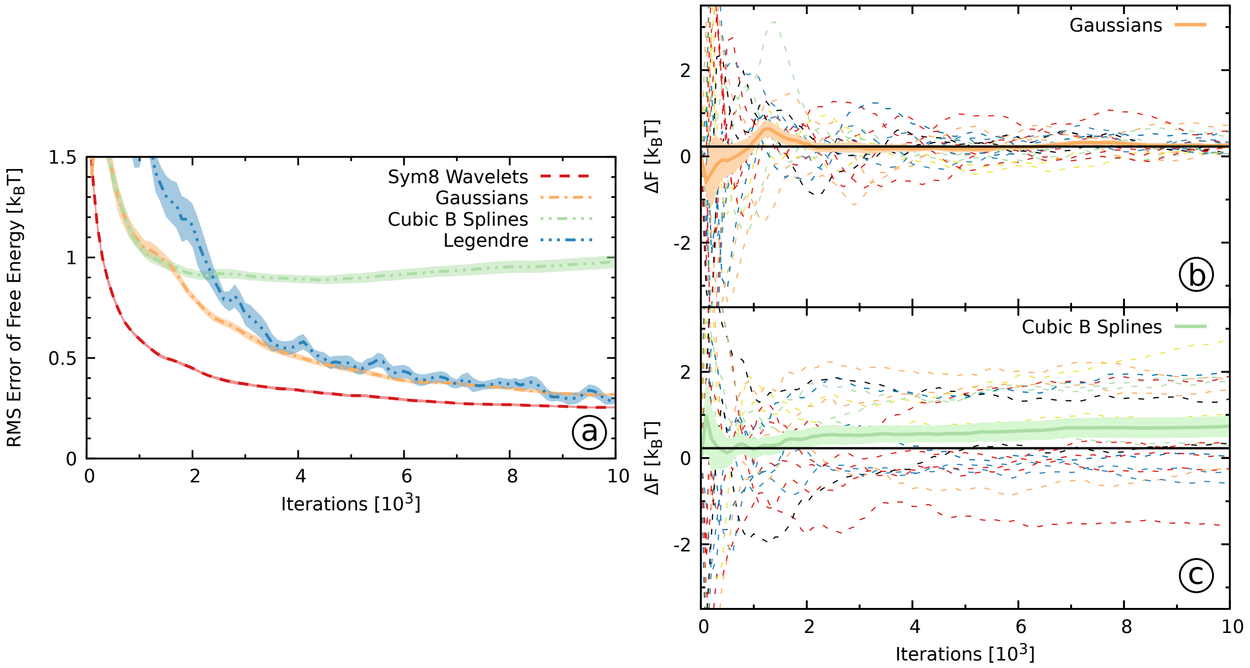

Figure S7: (**a**) The RMS Error of the FES calculated directly from the bias potential for the Wolfe-Quapp system (see Sections 3.2 and 4.1 in main text) as a function of the number of bias iterations for different basis functions. The lines denote the average over 20 runs and the shaded area the corresponding standard error. (**b**,**c**) Time evolution of the free energy difference estimate for simulations with Gaussians and cubic B-splines, analogue to Figure 3 in the main text. The dashed lines show the result for the individual simulation, while the solid line denotes the average and standard error.

Figures S7**b**,**c** show additional calculations of free energy difference between the two states of the the Wolfe-Quapp potential when using Gaussians and cubic B-splines. Because the simulations with cubic B-splines did not converge to the correct free energy surface, the calculated free energy differences are also not correct. In comparison to the figures given in the main text, the Gaussian basis functions give a better approximation of the free energy difference than Legendre polynomials but yield slightly worse results than the Sym8 wavelets.

In Figure S8**a**, we give the error measure of the FES for the rotated Wolfe-Quap potential. We note that although this is a two-dimensional potential, we biased only the x-direction.

The error measure therefore compares the one-dimensional FES received from the bias to the projection of the reference FES on the x-direction. After an initial phase of about 2,000 iterations, the RMS error from simulations with Gaussians and cubic B-splines are not changing fundamentally anymore but only fluctuating around a value of about 1.5 $k_\mathrm{B}T$. On the other hand, simulations with Legendre polynomials and Sym8 wavelets are still improving their respective FES approximation after the initial phase. The approximation from the wavelet simulations is the overall best. It fluctuates only minorly and has the lowest average error.

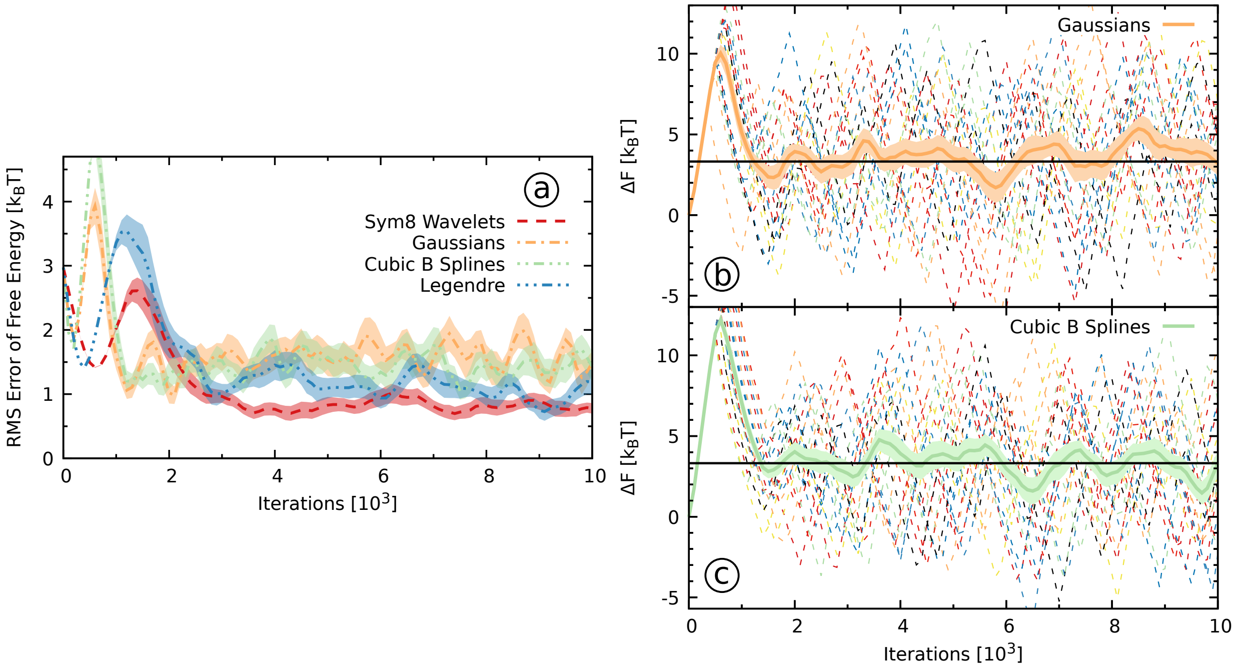

Figure S8: (**a**) The RMS Error of the FES calculated directly from the bias potential for the rotated Wolfe-Quapp system (see Sections 3.3 and 4.1 in main text) as a function of the number of bias iterations for different basis functions. The lines denote the average over 20 runs and the shaded area the corresponding standard error. (**b**,**c**) Time evolution of the free energy difference estimate for simulations with Gaussians and cubic B-splines, analogue to Figure 4 in the main text. The dashed lines show the result for the individual simulation, while the solid line denotes the average and standard error.

In Figures S8**b**,**c** we show additional plots of the calculated free energy differences between the two states of the rotated Wolfe-Quapp potential for Gaussians and cubic B-splines. There are only minor differences between the results from the two basis functions. Although we

can see that they yield approximately the correct value, the results are not very precise and we conclude an overall worse performance compared to the simulations with Sym8 wavlelets and Legendre polynomials shown in the main text.

S5 Calcium Carbonate Association: Collective variables

For the simulation of the association of the calcium and carbonate ion we use two collective variables:

1) The distance between the calcium atom and the carbon of the carbonate ion.

2) The coordination number of the calcium atom with the surrounding water molecules.

In practice the coordination number is calculated using a switching function

$$\sum_i s_i = \sum_i \frac{1 - \left(\frac{r_i - d_0}{r_0}\right)^n}{1 - \left(\frac{r_i - d_0}{r_0}\right)^m} \tag{S2}$$

where the index i runs over all oxygen atoms of the water molecules and r_i denotes the distance from the atom to the calcium atom. The parameters of the switching function are set to the same values as in ref S3, that is, $r_0 = 1.0$ Å, $d_0 = 2.1$ Å, $n = 4$, $m = 8$.

S6 Calcium Carbonate Association: Projections of Free Energy Surfaces

For each of the nine simulation runs conducted for the calcium carbonate system (three for each biasing method: VES with wavelets, VES with Chebyshev polynomials, well-tempered metadynamics) we calculate estimates of the free energy surface in two different ways: 1) directly from the bias potential, 2) via reweighting of the trajectories. Here, we will present

plots of the free energy as a function of the distance between the ions (the first CV). For each biasing method, one exemplary estimate that was obtained directly from the bias was already given in Figure 5**a** of the main text. Figure S9 provides the comprehensive view on all the sets, where each panel combines the estimates from the three different independent runs of a biasing method. The left column shows the estimates obtained directly from the bias potential, while the right column shows the reweighted results. Additionally the three reference FES of Ref. S3 are given. We observe that the estimates from the three

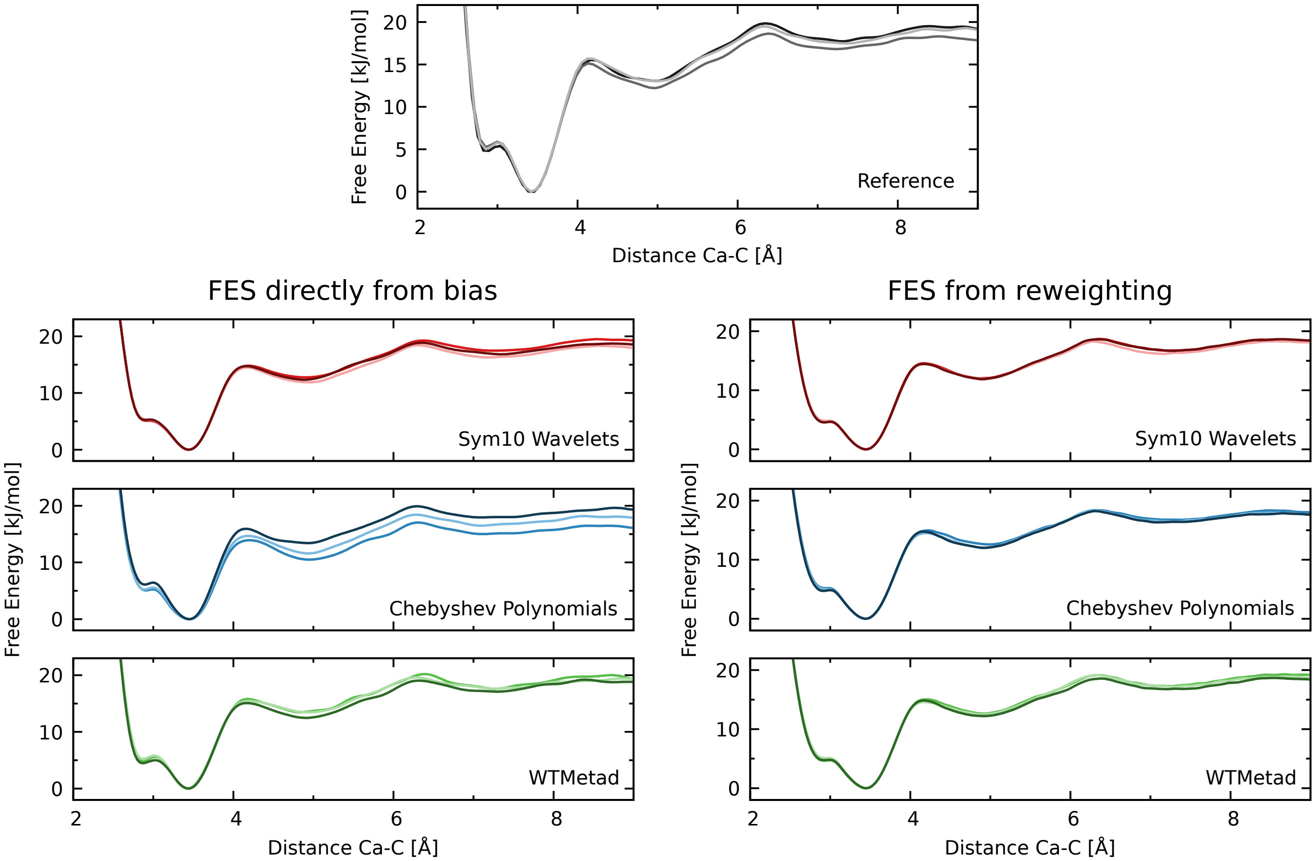

Figure S9: FES as a function of the ion distance for the calcium carbonate system.
Top panel: Reference FES obtained from Ref. S3.
Left column: FES estimates directly from the bias potential for three runs each of Sym10 wavelets, Chebyshev polynomials and Metadynamics simulations.
Right column: FES estimates obtained by reweighting of the trajectories for the same runs.

independent runs differ only marginally for all sets. The biggest differences are visible for the estimates directly from the bias, and there most prominently for the simulations with

Chebyshev polynomials. There, the different estimates differ on the order of several kJ/mol at the unbound state. For the other two datasets directly from the bias, the WTMetad runs also differ slightly over the full range, while small differences for the wavelet simulations are only visibly in the undbound region. Looking at the reweighted estimates in the right column of Figure S9, all data sets of each biasing methods agree very closely.

S7 Calcium Carbonate Association: Free Energy Difference

Here we give the numerical values of the free energy differences calculated in Section 4.2 and shown in panels **c**,**d** of Figure 6 in the main text.

The values of the individual runs were calculated by taking the last nanosecond of the simulation. We then took in total 100 values spaced every 10 ps and calculated the average as well as the standard deviation. Note that we take the standard deviation as quantity of uncertainty here, because the averaging is not over uncorrelated measurements but over correlated time series. Therefore, we treat the averaged result from each individual simulation as one uncorrelated measurement and give the standard deviation as uncertainty for obtaining this value.

For comparison with other works, we also calculate the free energy differences of the reference data obtained from Ref. S3, which can be seen at the top of Figure S9. Because we do not have the time evolution of the FES in this case, we can only obtain a single value without uncertainty for each run.

Finally, we also combine the values of the three individual runs of each data set by averaging and calculate the respective standard errors, while neglecting the uncertainties of the individual runs. All values can be found in Table S1.

Clearly visible is the lower uncertainty of the reweighted data sets, which corresponds to less fluctuations of the FES estimate when obtaining it by reweighting. Nevertheless, the

Table S1: Calculated values of ΔF for the different simulations. All values are given in units of kJ/mol. For every simulation, the values were calculated from the average of the last nanosecond both directly from the bias as well as via reweighting. The uncertainty of the runs is the standard deviation of the values of the last nanosecond. We only have a single value per run for the reference data and can therefore not give an uncertainty in this case. For the average the given uncertainty is the standard error from the combination of the three runs, where we neglected the uncertainties of the individual runs.

	run 1	run 2	run 3	average
Sym10 wavelets (bias)	9.2 ± 0.2	8.81 ± 0.06	9.32 ± 0.09	9.1 ± 0.2
Sym10 wavelets (reweighted)	9.08 ± 0.06	9.12 ± 0.04	9.21 ± 0.03	9.13 ± 0.04
Chebyshev polynomials (bias)	9.2 ± 0.6	8.9 ± 0.3	8.7 ± 0.6	8.9 ± 0.1
Chebyshev polynomials (reweighted)	9.4 ± 0.1	8.87 ± 0.03	8.62 ± 0.07	9.0 ± 0.2
WTMetad (bias)	10.0 ± 0.2	9.6 ± 0.5	9.9 ± 0.5	9.8 ± 0.1
WTMetad (reweighted)	9.79 ± 0.04	9.4 ± 0.1	9.38 ± 0.04	9.5 ± 0.1
Reference	9.94	9.08	9.78	9.6 ± 0.3

individual values differ a lot between the different methods and also the individual runs for the data obtained with Chebyshev polynomials or WTMetad. The values obtained from reweighting the simulations with Sym10 wavelets differ only slightly between the runs.

References

(S1) Demuynck, R.; Rogge, S. M. J.; Vanduyfhuys, L.; Wieme, J.; Waroquier, M.; Van Speybroeck, V. Efficient Construction of Free Energy Profiles of Breathing Metal—Organic Frameworks Using Advanced Molecular Dynamics Simulations. *J. Chem. Theory Comput.* **2017**, *13*, 5861–5873.

(S2) Kingma, D. P.; Ba, J. Adam: A Method for Stochastic Optimization. 3rd International Conference on Learning Representations. 2015.

(S3) Raiteri, P.; Demichelis, R.; Gale, J. D. Thermodynamically Consistent Force Field for Molecular Dynamics Simulations of Alkaline-Earth Carbonates and Their Aqueous Speciation. *J. Phys. Chem. C* **2015**, *119*, 24447–24458.
